# Evolution of Pairing Orders between Pseudogap and Superconducting Phases of Cuprate Superconductors

**DOI:** 10.1038/s41598-018-38288-7

**Published:** 2019-02-08

**Authors:** Wei-Lin Tu, Ting-Kuo Lee

**Affiliations:** 1 Department of Physics, National Taiwan University, Daan, Taipei 10617 Taiwan; 2 Laboratoire de Physique Théorique, IRSAMC, Université de Toulouse, CNRS, UPS, Toulouse, France; 3 Institute of Physics, Academia Sinica, Nankang, Taipei 11529 Taiwan

## Abstract

One of the most puzzling problems of high temperature cuprate superconductor is the pseudogap phase (PG) at temperatures above the superconducting transition temperature in the underdoped regime. The PG phase is found by the angle-resolved photoemission spectra (ARPES) to have a gap at some regions in momentum space and a fraction of Fermi surface remained, known as Fermi arcs. The arc turns into a d-wave SC gap with a node below the SC transition temperature. Here, by studying a strongly correlated model at low temperatures, we obtained a phase characterized by two kinds of pairing order parameters with the total momentum of the Cooper pair to be zero and finite. The finite momentum pairing is accompanied with a spatial modulation of pairing order, i.e. a pair density wave (PDW). These PDW phases are intertwined with modulations of charge density and intra-unit cell form factors. The coexistence of the two different pairing orders provides the unique two-gaps like spectra observed by ARPES for superconducting cuprates. As temperature raises, the zero-momentum pairing order vanishes while the finite momentum pairing orders are kept, thus Fermi arcs are realized. The calculated quasiparticle spectra have the similar doping and temperature dependence as reported by ARPES and scanning tunneling spectroscopy (STS). The consequence of breaking symmetry between x and y due to the unidirectional PDW and the possibility to probe such a PDW state in the PG phase is discussed.

## Introduction

A long-standing unresolved puzzle of the cuprate high temperature superconductors is the nature of pseudogap (PG) phase^1,2^. Below the PG temperature T* there are experimental evidences of breaking some crystalline symmetry^3,4^. Breaking of time-reversal symmetry with observation of intra-cell magnetic moments has also been reported^5^. Many more new evidences suggest that this phase should be a nematic phase that breaks the four-fold rotation symmetry of the copper oxygen lattice^6–8^. In particular there are many reports of the charge density waves (CDW) or spin density waves (SDW) in the SC and PG phases^9–13^. Some of these are likely unidirectional hence without four-fold rotation symmetry. There are experimental evidences indicating the presence of fluctuating or short-range-ordered CDW in the PG phase^12,14,15^. Furthermore these CDW orders were also observed in the superconducting (SC) phase^14,16–20^. Once the CDW sets in and breaks four-fold symmetry^21^, the symmetry of pairing order in the SC phase of tetragonal crystal such as *Bi*_2_*Sr*_2_*CaCu*_2_*O*_8+*x*_ should not be expected as a pure *d*-wave as seen in experiments^22,23^. Thus the formation mechanism of these density waves and its relations with SC and pseudopgap phases are of great interests.

Before the discovery of these density wave orders in the cuprates, the PG phase has already posed a number of unexplained puzzles. Below a characteristic temperature T* but higher than the SC transition temperature *T*_*c*_, the excitation spectra showing a gap was first noticed by the relaxation rate of nuclear magnetic resonance^24^ and then by many other transport and spectroscopic measurements^25^. But the most direct observation of this gap structure was shown by the ARPES^26–28^. The energy-momentum structure shows an energy gap appears near the boundary, or the antinodal region, of the two-dimensional Brillouin zone (BZ) of the cuprate. However there are four disconnected segments of Fermi surface near the nodal region, or |*k*_*x*_| = |*k*_*y*_| = *π*/2. These segments called Fermi arcs have been reported to have their length shrink to zero^29,30^ when extrapolated to zero temperature. There are also results indicating that the arc length is not sensitive to temperature^14,27^. Then it could also be part of a small pocket^31,32^. This presence of finite fraction of Fermi surface is consistent with the Knight shift measurement^33^ showing a finite density of states (DOS) after the superconductivity is suppressed. The full Fermi surface is recovered either for temperature higher than T* or when doping increases beyond approximately 19% as the PG phase disappears. Below *T*_*c*_ the gap at antinode merges with the SC gap. Also the ARPES spectra at the antinodal region does not have the usual particle-hole symmetry associated with traditional superconductors. This asymmetric antinodal gap onsets at T* and it persists all the way to the SC phase^34,35^.

The phenomena of two gaps, one PG formed above the SC temperature *T*_*c*_ and additional SC gap below, and all the exotic behavior associated with it has attracted many attentions as discussed in recent reviews^34,36^. There are many theoretical proposals devoted to understand the PG as discussed in these review articles^1,37,38^. But so far it has been difficult to understand the temperature and doping dependence of the Fermi arcs, two gaps and other spectroscopic data, as well as its explicit relationship with the CDW orders and whether any of these are related with the Mott physics or the strong correlation.

However, there are growing evidences that these CDW are not a usual kind but are related to or could be a subsidiary order of the pair density wave (PDW). PDW is a state with spatial modulation of the pairing amplitude and it was first introduced by Larkin and Ovchinnikov^39^ and by Fulde and Ferrell^40^. The pairing order $< {C}_{{\rm{k}}+Q/2\uparrow }{C}_{-{\rm{k}}+Q/2\downarrow } > ={{\rm{\Delta }}}_{Q}({\bf{k}})$ has a <center of mass momentum *Q* and relative momentum **k**. Usual BCS pairing is for *Q* = 0, or Δ_0_(**k**). There were quite a number of works proposing that PDW state might be responsible for the many observed exotic phenomena^41–46^ in both SC and PG phases. Many of the works used phenomenological models and weak coupling approaches^47–50^, but some of the numerical works on microscopic models such as the Hubbard model and its low-energy effective *t* − *J* model, have found strong evidences for such a state or states. Actually there are many different kinds of PDW states that could be either unidirectional^51^ or bidirectional like a checkerboard, which are tabulated in ref.^52^ by us. For the unidirectional PDW state intertwined with CDW and SDW, so called the stripe state, was first proposed by the variational calculation for the *t* − *J* model^43^. It could have the same sign of *d*-wave pairing on each site or pairing is in-phase^52^ so that the period of modulation of pairing is same as charge density but only half of the SDW. Or it could be the anti-phase stripe having two domains with opposite pairing sign so that the period of pairing modulation is twice of the charge density. The in-phase stripe was later shown^53^ to be a stable ground state with half a hole in each period of CDW when a small electron-phonon interaction is included in the *t* − *J* model. This half-doped stripe may be what was observed in neutron scattering^9^ for the LBCO (*La*_2−*x*_*Ba*_*x*_*CuO*_4_) family.

The strongest theoretical support for the stripe state is from the recent numerical calculation by Corboz *et al*.^54^. By using the infinite projected entangled-pair states (iPEPS) method, they found that the *t* − *J* model has several stripe states, with nearly degenerate energy as the uniform state. Even though the method has demonstrated its ability to obtain the lowest energies than all previous numerical methods, they still cannot pinpoint which state is the ground state. The results are quite consistent with the most recent numerical studies on the Hubbard model^55^. They found the stripe states have lower energies than the uniform SC state at 1/8 hole density and for *U*/*t* = 8 and 12. The period of the PDW moves toward 4 or 5 lattice spacing as *U* increases and this is more in line with result of the *t* − *J* model.

Besides the stripe state there is also the possibility of a PDW state coupled with CDW only and without SDW involved. Capello *et al*.^56,57^ have proposed such a state with an uniform pairing order but it is not a pure *d*-wave order. Instead of proposing a possible state by conjecturing, we have solved a set of self-consistent equations derived from the renormalized mean-field theory (RMFT)^51,52^ based on the generalized Gutzwiller approximation (GWA)^58^. Of the many low energy solutions we found, there is a PDW state coupled with a uniform *d*-wave pairing order that explains a number of properties measured by the scanning tunneling spectroscopy (STS) on BSCCO (*Bi*_2_*Sr*_2_*CaCu*_2_*O*_8+*x*_) and NaCCOC (*Ca*_2−*x*_*Na*_*x*_*CuO*_2_*Cl*_2_)^59^. This particular state, referred to as nodal pair density wave (nPDW)^52,60^, has pairing orders Δ_*Q*_(**k**), Δ_−*Q*_(**k**), for several *Q* and also an uniform Δ_0_(**k**) that produces a nodal like local density of states (LDOS). The period of the CDW is about half of the PDW. Furthermore, by including the Wannier function in our calculation to take into account the effect of oxygens that were neglected in the simple *t* − *J* model^60^, we are able to compute the continuum local density of states of the nPDW. The energy dependence of intra-unit cell form factors and spatial phase variations of these states agrees remarkably well with the STS experiments^59,61^. The success of this nPDW state to quantitatively explain the real space spectra measured by STS in the SC phase naturally leads us to study the spectra in momentum space measured by ARPES.

Instead of concentrating on the microscopic models, the Landau-Ginzburg free energy formalism is used to study the intricate relationship between PDW, CDW, and the uniform pairing order^44–46,62,63^. By including phases of PDW, they could discuss vortex and dislocations as well as the phase diagram. They pointed out that PDW could be responsible for the PG phase. Some of the properties we shall discuss below are consistent with their results; however, they did not consider bond order as an independent field whereas we have shown^52^ that bond order with dominant intra-unit-cell form factor with *s*′ or *d* symmetry are associated with different PDW states such as stripes or nPDW, respectively. Neither are most of the phenomenological approaches^50,64^. Another interesting work by Lee^65^ proposed the Amperean pairing originated from the gauge theory of the resonating valence bond (RVB) picture as the main mechanism for the formation of PDW and it is the dominant order in cuprates. This theory prefers to have bidirectional PDW to have similar gaps at antinodes (*π*, 0) and (0, *π*). They also did not address the issue of bond orders.

In this paper the spectra associated with the nPDW state will be calculated first at *T* = 0 as our previous work but with emphasis on the energy-momentum dependence of the quasiparticles. Then we will extend our calculations to finite temperatures. The GWA used in the RMFT is considered to be a good approximation at zero temperature. The energy scale imposed by the strong Coulomb repulsion, or Hubbard *U*, is much larger than the scale of room temperature. In addition, both the two main “low” energy scales, *t* and *J* about 3000~4000 *K* and 1200 *K*, respectively, are also much larger. Hence we shall make an assumption that the GWA is reasonably accurate at low but finite temperatures.

After the RMFT is transformed to solve for the self-consistent equations at finite temperatures, we found the average or net uniform pairing order parameter (UPOP) of the nPDW state decreases to almost zero at a “critical” temperature *T*_*p*1_. This new state still has incommensurate modulations of charge density, pair density and bond orders intertwined, and we shall denote it as incommensurate pair-density-wave (IPDW) state. Just as nPDW state this IPDW state also has the dominant intra-unit-cell *d*-form factors^52,60^ and particle-hole asymmetry for the ARPES spectra^35^ at the antinodal region. The major difference with nPDW is the appearance of Fermi arcs and a substantial increase of DOS at Fermi energy but without UPOP. As temperature further increases to *T*_*p*2_, there is no longer a solution of this state. The value of *T*_*p*2_ increases sharply as doping is reduced. The DOS at Fermi energy increases only slightly between *T*_*p*1_ and *T*_*p*2_. The DOS also increases slightly with increasing doping. Comparing these results with experimental data on ARPES^34,35^ and DOS deduced from Knight shifts^33^, we conclude that it is quite reasonable to take *T*_*p*1_ as the SC transition temperature *T*_*c*_ and *T*_*p*2_ as a mean-field version of the PG temperature T* of the copper oxides. These issues will be discussed after the results are presented.

## Results and Discussions

All our results reported here are for the two-dimensional *t* − *t*′ − *J* model shown in Eq. (1), on a square lattice:

1 $$\begin{array}{l}H=-\,\sum _{i,j,\sigma }\,{P}_{G}{t}_{ij}({c}_{i\sigma }^{\dagger }{c}_{j\sigma }+H.\,C.\,){P}_{G}+\sum _{\langle i,j\rangle }\,J{S}_{i}\cdot {S}_{j}\end{array}$$

where nearest neighbor hopping *t*_*ij*_ = *t* where *ij* is set to be the nearest neighbor sites and *J* is set to 0.3*t*. ${P}_{G}={\prod }_{i}\,(1-{n}_{i\uparrow }{n}_{i\downarrow })$ is the Gutzwiller projection operator, while ${n}_{i\sigma }={c}_{i\sigma }^{\dagger }{c}_{i\sigma }$ stands for the number operator for site *i*. Spin *σ* is equal to ±. *S*_*i*_ is the spin one-half operator at site *i*. If *i* and *j* are nearest neighbor (next nearest neighbor), *t*_*ij*_ = *t* (*t*_*ij*_ = *t*′ = −0.3*t*). The effect of the projection operator *P*_*G*_ is treated with a mean-field GWA; we replace the the constraint of forbidding the double occupancy of two electrons on the same site with Gutzwiller factors. Thus one can follow RMFT^51^ to find the various low energy states at *T* = 0 as we have done in our previous works^52,60^ focused on studying the STS. We shall first use these results to calculate the quasi-particle Green’s functions and its spectra density. Then we will generalize the calculation to finite temperatures by assuming the Gutzwiller factors remain unchanged as temperature is slightly raised. Discussion about the calculation is presented in the Methods section.

Usually in our theory there are four intertwined mean-field order parameters: local AF moment ${m}_{i}^{v}$, pair field ${{\rm{\Delta }}}_{ij\sigma }^{v}$, bond order ${\chi }_{ij\sigma }^{v}$, and hole density *δ*_*i*_, where *i* is a site position and *ij* is the nearest neighbor bond, but in this paper we will not consider ${m}_{i}^{v}$. By including the Gutzwiller factors in the *t* − *t*′ − *J* model, the mean field Hamiltonian can be written as the BdG equations. After it is diagonalized, iterative method is used to achieve self-consistent solutions. If we are only interested in unidirectional density waves with modulation in x direction, we could exploit the translational invariance in y direction by switching the site index *i* = (*i*_*x*_, *i*_*y*_) to (*i*_*x*_, *k*_*y*_) by taking the Fourier transform in *i*_*y*_ as shown in ref.^60^, and this makes the numerical calculations much more efficient. As shown in refs^52,60^, there are many nearly energy degenerate solutions with commensurate or quasi-incommensurate modulations with several periods mixed together. The *d*-wave pair field at each bond could have the same sign or they have domains with opposite signs. Most of the inhomogeneous solutions have PDW intertwined with CDW and bond order. But if doped hole concentration is low in the very underdoped region, there are also solutions with spin density wave (SDW) intertwined with the other three orders. Readers can find detail discussions about these states and also bidirectional checker board solutions in ref.^52^. Here we will only consider nonmagnetic solutions.

### Particle-hole asymmetry

We will first discuss ARPES spectra for nPDW states^60^ that have been shown to provide quantitative agreement with STS experiments^59^ on BSCCO and NaCCOC. These states with incommensurate PDW, CDW, and bond order wave coexisting have a UPOP exhibiting a *d*-wave nodal like LDOS at low energy. Their energy dependence of the intra-unit-cell form factors with *s*, *s*′ and *d* symmetry and the spatial phase difference agree well with the STS experiments. The characteristics of a particular nPDW state obtained at hole concentration 0.125 for a 32 × 32 lattice is quite similar with our previous works in^52,60^. Figure S1 in the supplementary material (SM) shows the variation of hole density and pairing order parameter as a function of positions (Fig. S1(a)) and their Fourier transform (Fig. S1(b)), the LDOS at several selected sites (Fig. S1(c)), and the intra-unit-cell form factors for three symmetries (Fig. S1(d)). As shown in Fig. S1(b), The state has various periods mixed but mostly a period close to 4*a* in the x-direction for charge density modulation and 8*a* for pairing order modulation.

**Figure 1:**
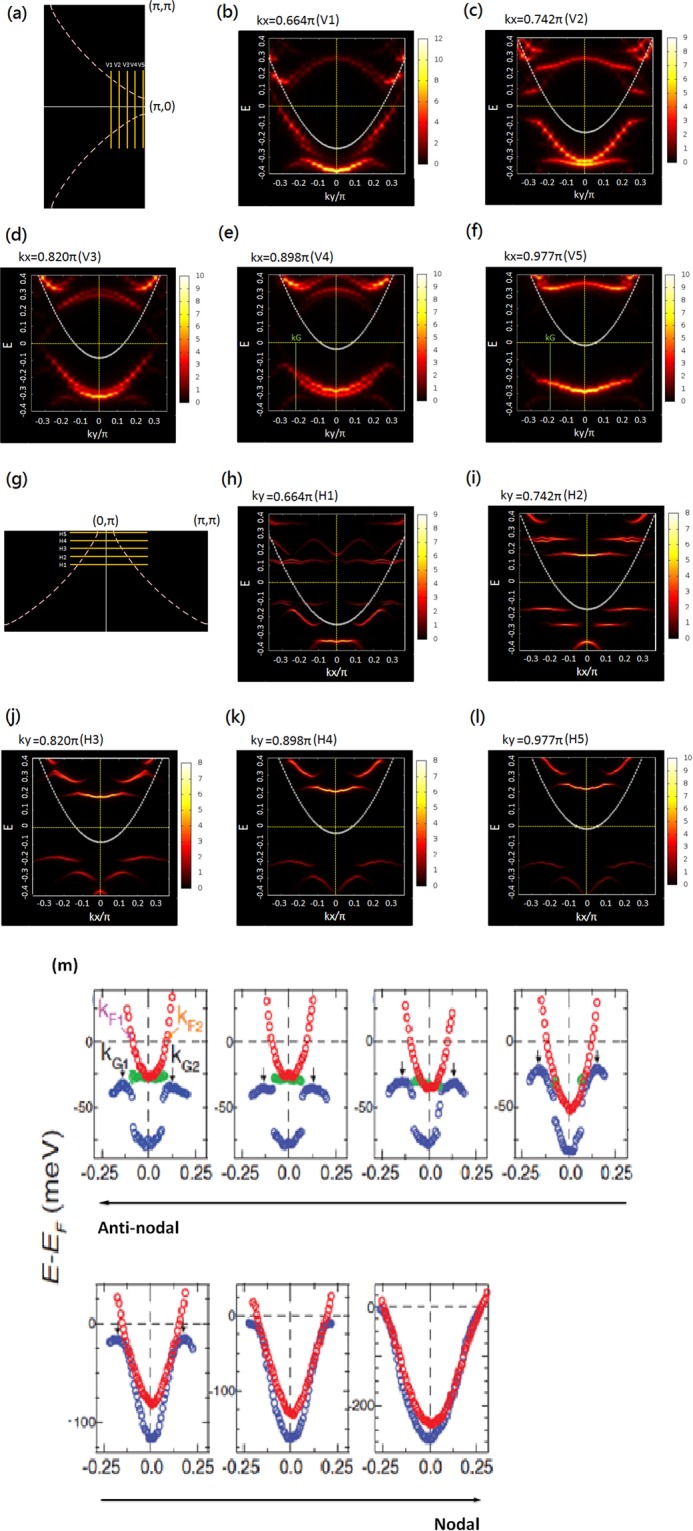
The quasiparticle spectra of a nPDW state calculated in a 32 × 32 lattice for hole concentration 0.125: (**a**) the vertical cuts (*V*1–*V*5) denote the y component of the momentums scanned from (**b**) (near nodal region) to (**f**) (anti-nodal region). (**b**–**f**) Quasiparticle spectra weight for each cut as a function of *k*_*y*_ with a fixed *k*_*x*_ value shown above each figure. (**g**) The horizontal cuts (*H*1–*H*5) denote the x component of the momentums scanned from (**h**) (near nodal region) to (**l**) (anti-nodal region). (**h**–**l**) Quasiparticle spectra weight for each cut as a function of *k*_*x*_ with a fixed *k*_*y*_ value shown above each figure. (**m**) Spectra figures from the ARPES results^35^ for comparison. Notice that the sequence of showing these figures goes from the anti-nodal to nodal direction, which is different from that of 1(**b–f**).

The spectral density *A*(*k*_*x*_, *k*_*y*_, *ω*) of the above state is calculated by using Eq. (5) at *T* = 0. We choose the width Γ = 0.01*t* unless specifically mentioned otherwise. In Fig. 1(a) we scan the momentum space near the antinodal region (*k*_*x*_, *k*_*y*_) = (*π*, 0) by having 5 vertical cuts (*V*1–*V*5) perpendicular to the *k*_*x*_ axis. The energy dependence of the spectral weight as a function of the y component of the wave vector (*k*_*y*_) for the five cuts are shown in Fig. 1(b–f). A very striking result is the complete lacking of particle-hole symmetry in the spectra as a BCS theory would have predicted. The white curves denote the dispersion of a uniform Fermi-liquid state (FLS) without pairing at dopant density 0.125. The curve crossing zero energy are Fermi momenta *k*_*F*_. The five cuts show that near the nodal region *V*1 the gap at *k*_*F*_ is small and increases substantially approaching toward the antinodes *V*5. Most interesting part is the dispersion along each cut bends back after passing the minimum energy gap, which is determined by looking at bands above and below Fermi energy. These back-bending momentum *k*_*G*_ moves away from *k*_*F*_ as momenta approaching antinodes. The momentum *k*_*G*_ is determined by using the energy distribution curves (EDCs). Details are discussed in SM. For the ARPES experiment, as demonstrated in Fig. 1(m), only the occupied states or the states with negative energies are measured; hence it cannot show the momenta with minimum gap but it can determine the back-bending momenta *k*_*G*_. Indeed our result is very consistent with the experiments^35^ showing this particle-hole asymmetry which is very different from usual BCS superconductors that *k*_*G*_ = *k*_*F*_. It was first pointed out by Lee^65^ that the difference between *k*_*F*_ and *k*_*G*_ and the way two approaches each other near nodal region is inconsistent with pure CDW either.

In the experiment, the spectra along *k*_*x*_ direction is same as in *k*_*y*_. This is likely due to the sample packed with x- and y- oriented short-range ordered unidirectional domains as seen in STS^59^. But here we only have one unidirectional nPDW, hence spectra along *k*_*x*_ and *k*_*y*_ are different. In Fig. 1(g), we scan five horizontal cuts (*H*1–*H*5) from near nodal region (h) to antinodal region (l). Comparing 1(b) with 1(h), we can see that there is no state at low energy for 1(h). In general the minimum gaps are the same for *H*5 cuts near (0, *π*) and *V*5 cuts near (*π*, 0). It seems that the gap does not change much from 1(j) to 1(l) while it increases significantly from 1(d) to 1(f). The occupied bands are quite flat along the *k*_*x*_ direction near (0, *π*), and hence it is difficult to determine the bending vectors *k*_*G*_. This kind of spectra near (0, *π*) for a *x*-directional PDW is quite different from what one would expect for a pure CDW^65^. We also note that energy gap value of 0.21*t* at the parallel direction (Fig. 1(i)) with respect to the modulation direction of density waves is about the same as in the perpendicular direction (Fig. 1(f)). This will be discussed further in the discussion section.

We have also considered anisotropy^51^ in hopping *t*_*x*(*y*)_ and *J*_*x*(*y*)_ and if we decrease *t*_*x*_ with respect to *t*_*y*_ (${J}_{x}/{J}_{y}={t}_{x}^{2}/{t}_{y}^{2}$), then the nPDW state modulated in x-direction will no longer have a pure *d*-wave but a *s*′ + *d* wave. The energy gap determined by the *H*5 cut near (0, *π*) increases but the value of *V*5 cut near (*π*, 0) is reduced. We will discuss this further in the Discussion section later.

### Two Gaps in the SC phase

**Figure 2:**
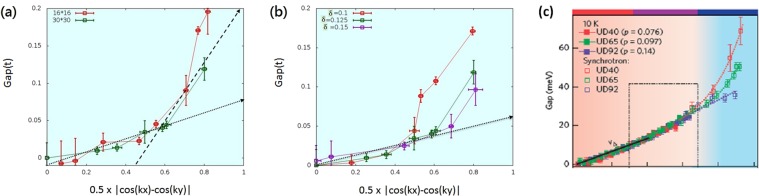
The gap value evolving from nodal to anti-nodal region for nPDW for (**a**) two different lattice sizes at doping 0.125; (**b**) different doping levels but same size (30 × 30). Red, green and purple lines are just guides for the eyes. The black dotted (dashed) line is a fitted pure d-wave (antinodal) gap with gap size about 0.075~0.08*t* (0.2*t*). The Gaussian width used here is Γ = 0.01*t*. (**c**) Gap behavior from ARPES within the superconducting dome^34^.

The quasi-particle spectra in Fig. 1 also show an energy gap increasing as momentum approaches antinodal region. To compare with the result of ARPES experiment we shall use the EDCs to determine the gap. By taking a scan along a linear cut near the Fermi surface (the white curves in Fig. 1(a)), we can determine the k value that has the smallest energy difference from chemical potential. This will determine the energy gap at this *k* value. Going from the nodal direction at *k*_*x*_ = *k*_*y*_ to the antinodal region (*k*_*x*_, *k*_*y*_) = (*π*, 0), the energy gap is plotted as a function of |*cos*(*k*_*x*_) − *cos*(*k*_*y*_)|/2 in Fig. 2 for nPDW states. In Fig. 2(a), at hole concentration 1/8, the red curve is obtained for a 16 × 16 lattice and green for 30 × 30. The vertical error bars are either determined by the width of the peak or by average of two nearby peaks with nearly the same magnitude. The horizontal error bars are due to the effect of discrete k values. The results are essentially the same for these two different lattice sizes and in fact, there is also not much difference with the solution of 60 × 60 lattice presented in^60^. The vertical error bars are only slightly larger for the smaller 16 × 16 lattice. The slope of both curves increases as the momentum gets closer to the antinode. The dotted line in Fig. 2(a) indicates the linear fitting of a pure *d*-wave pairing gap (Δ_*c*_|*cos*(*k*_*x*_) − *cos*(*k*_*y*_)|/2) near the nodal region. The fitted gap value Δ_*c*_ is much smaller than the gap at antinodal direction. The dashed line indicates a second gap near the antinodal region. Thus we have two different *d*-wave gaps near the node and antinode.

Furthermore we can examine the variations of these gaps with dopant concentration. In Fig. 2(b) the gaps are plotted for nPDW states calculated for a 30 × 30 lattice for three hole concentrations: red for *δ* = 0.1, green for 0.125 and purple for 0.15. As hole concentration decreases, the gap at the antinodal region gets larger while deviation from the dotted line starts closer to the nodal *k*. The value of the gap at antinode could reach 0.2*t* or about 80 *meV* just as in the experiments^34^. At larger doping these two *d*-wave gaps seem to approach each other as a single gap which is expected in the usual BCS state. Due to the finite size effect, we cannot determine if the *d*-wave like gap for the three hole concentrations in Fig. 2(b) are exactly the same, but it looks close enough and with a value about 0.08*t* ~ 32 *meV* for *t* = 0.4 *eV*. In Fig. 2(a,b) we used a constant Γ = 0.01*t*, but the result is insensitive to the choice of the Γ in calculating the spectra density. This is very consistent with ARPES results shown in refs^34,66^ and we put alongside the figure of gap behavior of superconducting state from ref.^34^ in Fig. 2(c) for comparison. They found the gap value about 39 *meV* near the nodal region for several different hole concentrations.

So far we have discussed the gaps and spectral density of the superconducting nPDW states that were discussed previously in refs^52,60^. The state is quasi-periodic in the sense that it has several periods mixed but the dominant one is near 4*a*. However it was recently proposed^67^ that the charge density modulation could be strictly 4*a* but the phase could jump with multiple values of 2*π*/4 from its original perfect periodic values. This is the CDW with phase “discommensuration” (DC) as defined by McMillan^68^. In the SM, Fig. S2 shows a nPDW state with 4*a* charge density modulation and phase DC for a 36 × 36 lattice at doping 0.125. The energy per site has the same value as the previous nPDW state. Almost all the properties are very similar with the nPDW states discussed before^52,60^. The intra-unit-cell form factors are dominated by *d*-symmetry. The pair density modulation is mostly dominated by a vector *Q*_*p*_, and the CDW has mainly a peak at 2*Q*_*p*_ and also a peak at *Q*_*p*_. We will not discuss it here and detail can be found in SM.

### Finite temperature IPDW states

All the states discussed above are SC with a net *d*-wave UPOP. These were obtained by solving the mean-field BdG equations self-consistently at *T* = 0. This also implies that we have a finite Δ_0_(**k**) as UPOP is same as average of the product of (*cos*(*k*_*x*_) − *cos*(*k*_*y*_)) and Δ_0_(**k**). We recall that the strong correlation effect of Mott physics is originated from the very large on-site Coulomb repulsion or Hubbard *U*. This effect is translated into a Gutzwiller projection operator to prohibit double occupancy of electrons at each lattice site in the *t* − *t*′ − *J* model (Eq. (1)). By following the GWA^58^, we replace these projection operators by Gutzwiller factors, which are functions of hole density. Here we will make an intuitive assumption that these Gutzwiller factors remain unchanged at temperatures much smaller than the relevant energy scale *t* and *J* which are of order 0.4 *eV* and 0.12 *eV*, respectively. Thus the BdG equations are easily generalized to finite temperatures and we could again find self-consistent solutions at finite *T*. Details are discussed in the Methods section. Here we will present the results.

**Figure 3:**
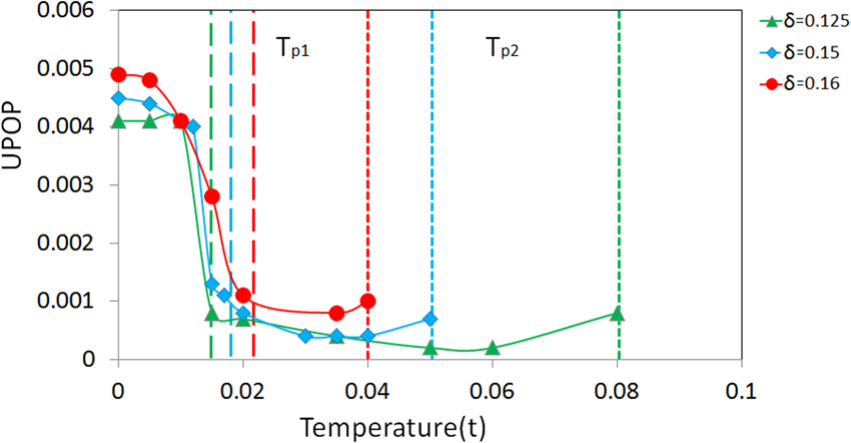
UPOP vs temperature for *δ* = 0.125, 0.15, and 0.16. *T*_*p*1_ and *T*_*p*2_ for each case are marked with different dotted lines of the same colors. The lattice size is 30 × 30.

In Fig. 3, the average or net UPOP calculated for a lattice of 30 × 30 with doping 0.125, 0.15, and 0.16 are plotted as a function of *T*/*t*, shown in green, blue and red marks. *T*_*p*1_ and *T*_*p*2_ for the three hole concentrations are also denoted. For simplicity *T*_*p*1_ is determined when the magnitude of UPOP reaches about 0.001. The three curves are quite similar except that near *T* = 0, *x* = 0.16 has the largest pairing order and also the largest value of *T*_*p*1_; however its *T*_*p*2_ is the smallest. The meaning of *T*_*p*2_ where no PDW exists becomes clearer when we examine its doping dependence. For *T* > *T*_*p*2_, the phase is actually a uniform *d*-wave state without modulations of charge and pairing. In previous work^52^, we already showed that the nPDW state and all other CDW or SDW states have a slightly higher energy than the uniform BCS state for the *t* − *J* and *t* − *t*′ − *J* model. If we include other interactions like long-ranged Coulomb interaction or a weak electron-phonon interaction^53,69^, these density wave states could become lower in energy. Even if we only consider *t* − *t*′ − *J* model, these nPDW states are stable solutions at local minimum, until they no longer exist at *T*_*p*2_ as shown in Fig. 3. Between *T*_*p*1_ and *T*_*p*2_ this new PDW state has essentially a zero UPOP or Δ_0_(**k**) = 0 but still finite Δ_*Q*_(**k**). It is an incommensurate PDW (IPDW) state, or the FFLO^39,40^ state, intertwined with modulation of charge density and bond orders.

**Figure 4:**
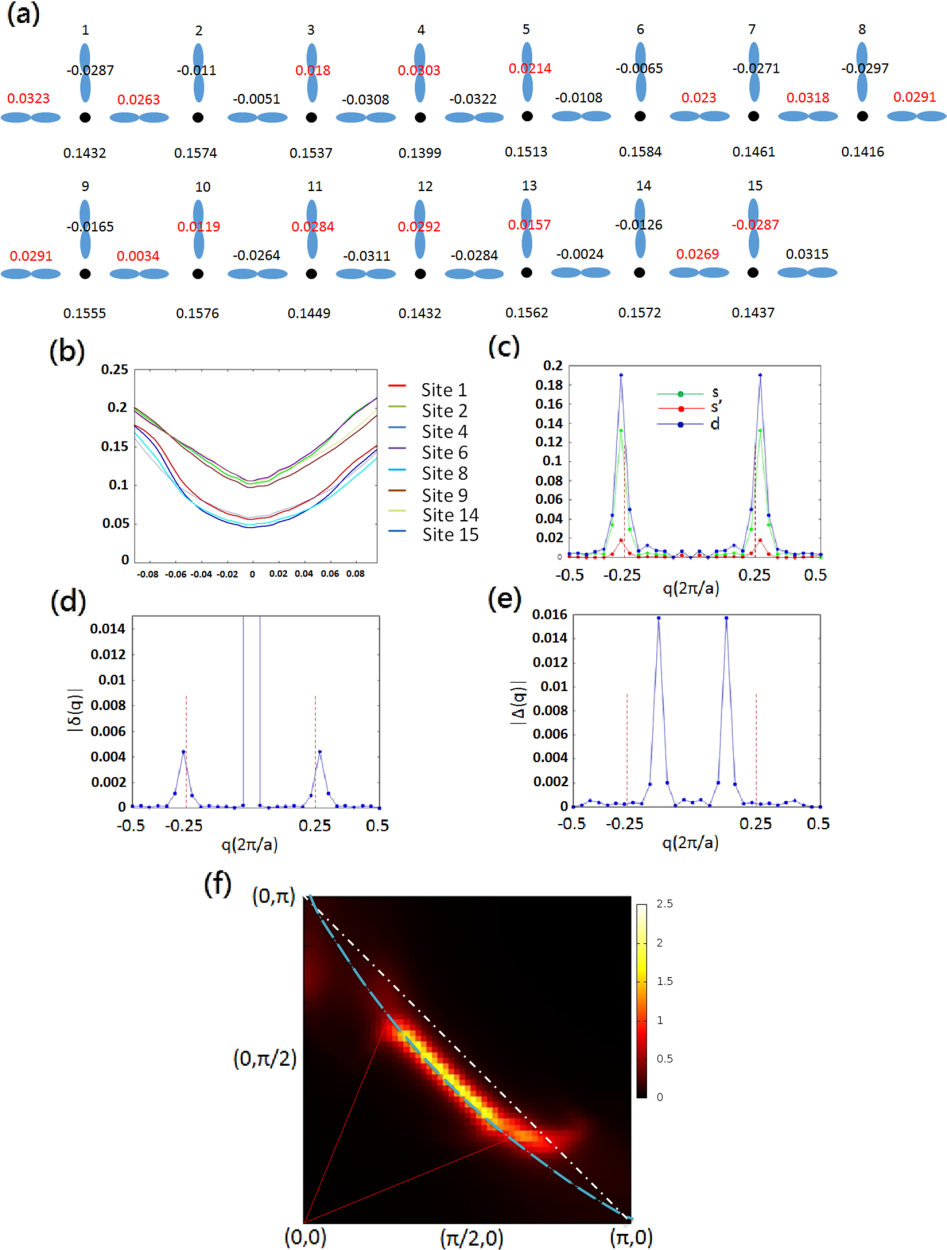
Properties of IPDW. (**a**) The real space modulation of IPDW. The red and black numbers on each bond denote the values of pairing order and the number at each site (black dots) is the hole density. (**b**) The LDOS for sites near the domain wall (2, 6, 9, 14 in 5(a)) and in the middle of nearby domain walls (1, 4, 8, 15 in 5(a)). (**c**) Different form factors and (**d**,**e**) Fourier transform of hole density (**d**) and pairing order (**e**). The red vertical dashed lines mark |*q*| = 0.5*π*/*a* corresponding to period 4*a*. Quasiparticle spectra with zero energy in k space for IPDW in 30 × 30 lattice sites at *T* = 0.035*t* are shown in (**f**) for *δ* = 0.15. The cyan dotted curve is the Fermi surface of Fermi liquid state with the same doping level. Γ used here is equal to $0.25\sqrt{{E}^{2}+{T}^{2}}$^71^.

The pattern of pairing order at each bond and hole density for an IPDW state with *δ* = 0.15 at *T* = 0.035*t* is shown in Fig. 4(a). The hole density is maximum at sites, e.g. 2, 6 and 10 at the pairing boundary where the paring order changes sign. The LDOS at a few selected sites are shown in Fig. 4(b). There is a finite constant LDOS near zero energy and it is not nodal like as the usual *d*-wave SC and nPDW states^52,60^. The Fourier transform of the intra-unit-cell form factor, hole density *δ*_*i*_ and pair field Δ_*ijσ*_ are shown in Fig. 4(c–e), respectively. the definition of form factors are^60^:

$$\begin{array}{l}d({\bf{q}})=FT({\tilde{\chi }}_{i,i+\hat{x}}-{\tilde{\chi }}_{i,i+\hat{y}})/2\\ s^{\prime} ({\bf{q}})=FT({\tilde{\chi }}_{i,i+\hat{x}}+{\tilde{\chi }}_{i,i+\hat{y}})/2\\ s({\bf{q}})=FT(1-{\delta }_{i})\end{array}$$

where *FT* refers to the Fourier transform and ${\tilde{\chi }}_{i,j}$ denotes the bond order (Eq. (3))^52^ with the mean value subtracted to emphasize the modulating component. $\hat{x}$($\hat{y}$) means one lattice displacement in x-(y-) direction. Both the modulation wave vector of the bond order wave and CDW, shown in Fig. 4(c,d), respectively, are 2*Q*_*p*_ = 0.52*π*/*a*, while the pairing modulation is dominated by *Q*_*p*_ = 0.26 *π*/*a* as shown in Fig. 4(e). Most of the properties of this IPDW state are similar with nPDW state except three distinctions: a negligible net UPOP, a finite Fermi arc as shown in Fig. 4(f) and *FT* of charge density has no peaks at *Q*_*p*_^45,46^. The color legend represents the spectral weight of these *k*-points on the arc in Fig. 4(f). Notice the arc is asymmetric with respect to exchanging *k*_*x*_ and *k*_*y*_ as the modulation along x-direction breaks the *x* and *y* symmetry. At the antinodal region the Fermi surface is gapped out similarly as the nPDW states.

**Figure 5:**
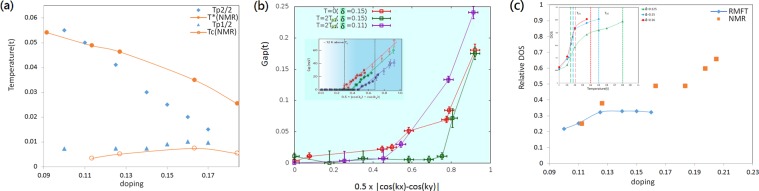
(**a**) Doping dependence of *T*_*p*1_ and *T*_*p*2_. *T*_*p*1_/2 and *T*_*p*2_/2 are shown with the blue triangles and diamonds respectively. The results from NMR^33^ are also shown for comparison. We choose 0.1*t* ~ 464 *K*. (**b**) The gap values scanned along the Fermi surface toward (*π*, 0) at *T* = 0 (red) and 2*T*_*p*1_ (green) for *δ* = 0.15 and 2*T*_*p*1_ (purple) for *δ* = 0.11. The inset shows the gap behavior of pseudo-gap phase from ARPES^34^. The meanings of symbols and colors are the same as Fig. 2(c). (**c**) Doping dependence of the relative DOS between IPDW and FLS (DOS_*IPDW*_/DOS_*FLS*_) at *T* = 0.035*t*. The experimental data from^33^ for *T* = 0 is also plotted for comparison. The inset shows DOS of IPDW vs temperature for *δ* = 0.125, 0.15, and 0.16. Γ we used here is $0.25\sqrt{{E}^{2}+{T}^{2}}$^71^.

In Fig. 5(a), *T*_*p*1_ and *T*_*p*2_ are plotted as a function of doped hole concentration with the blue triangles and diamonds respectively. We also plotted the PG phase temperature T* determined from the NMR measurement^33^ for *Bi*_2_*Sr*_2−*x*_*La*_*x*_*CuO*_6+*δ*_ in red color by taking *t* to be 0.4 *eV*. *T*_*p*1_ follows a dome shape and has a maximum at hole concentration 0.16. The steep suppression of *T*_*p*2_ with doping is similar with the PG temperature T*, and the values are also close if we reduce *T*_*p*2_ by about a factor of 2. This is not surprising as we have neglected the quantum fluctuation effect in this mean field theory^70^, and we also have assumed the Gutzwiller factors to have no *T* dependence. Also note that so far we have only considered long-range-ordered solutions and have neglected solutions of random x- and y- oriented domains with short-range IPDW states. Since IPDW state has a Fermi arc as shown in Fig. 4(f), we expect the gap should vanish at the Fermi surface near the nodal region. In Fig. 5(b) the gap value along the Fermi surface is plotted for *T* = 0 (red squares) and *T* = 2*T*_*p*1_(green squares). As a comparison, the IPDW state for 0.11 is also shown with the purple marks. Error bars are discussed in SM. This is very close to what is measured on BSCCO by ARPES^66^. The gap at antinode is essentially unchanged when the state changes from nPDW to IPDW. This is not surprising, since the antinode is still much larger than the temperature.

A very important property of the PG phase is the Knight shifts measured by NMR^33^; it shows that the DOS in the PG phase increases slowly with doping but is less than half of the DOS of the normal state for *T* > T* until it is near the critical doping about 0.2 where the PG phase disappears. It is also found that the Knight shift or DOS varies with temperature by less than 10% during the PG phase.

In the inset of Fig. 5(c), DOS is plotted as a function of temperature for three hole concentrations. Here we have assumed the width Γ, used in the spectra density calculation, is of the form ${\rm{\Gamma }}=0.25\sqrt{{E}^{2}+{T}^{2}}$^71^. The DOS is calculated at zero energy (within an energy range of ±0.004*t*) by averaging the LDOS at all sites. The DOS are all quite small and almost the same at *T* = 0 but it increases significantly at *T*_*p*1_. The DOS values between *T*_*p*1_ and *T*_*p*2_ increase with doping. This is likely due to the fact that the length of Fermi arc increases with doping. The variation of DOS with *T* between *T*_*p*1_ and *T*_*p*2_ for these three hole concentrations are also near 10% as in experiments. In Fig. 5(c), the ratio of DOS between the IPDW states and the FLS is plotted as a function of dopant concentration at *T* = 0.035*t*. Not only the doping dependence is very close to the experimental data^33^ shown as red symbols, the values are also close to the measured results. It is difficult for us to obtain solutions above dopant concentration 0.17 as *T*_*p*2_ and *T*_*p*1_ are very close (Fig. 5(a)). When the dopant concentration is above 0.18, we have no nPDW solution at *T* = 0 and no IPDW state at finite *T*. Thus we would recover the full Fermi surface and the relative DOS should be 1. For real materials this happens at concentration 0.2 instead of 0.18.

The quasi-particle spectra of the IPDW state is very similar with what is shown in Fig. 1 for nPDW state. The back-bending momentum *k*_*G*_ moves away from *k*_*F*_ approaching the antinode.

## Conclusion

Assuming that the Gutzwiller factors which take into account the renormalization effect of the strong correlation physics could have very small temperature dependence below room temperatures, we then generalize the renormalized mean-field theory to finite temperatures to study the prediction of the *t* − *t*′ − *J* model.

At low-temperature SC phase with a finite UPOP, a special self-consistent solution, the nPDW state first found by us in^52^, is shown to have the two *d*-wave pairing gaps as found by the ARPES. The smaller the doping, the larger is the gap magnitude at antinodes, but the nodal gaps are almost same for different dopings. The larger particle-hole asymmetry reported near the antinodal region is also well produced by the calculated spectra function. This nPDW state has a very special property that although it has a one-dimensional structure, the net pairing order or UPOP still has the four-fold *d*-wave symmetry. It is quite amazing that although the pairing value at each bond looks quite random (Fig. S1(a) in SM), its average has an exact *d*-wave symmetry. The spectra at antinodes (*π*, 0) and (0, *π*) are quite different but the values are close to each other (Gap values near the two antinodes my differ by 10% for different nPDW states. This is probably the accuracy of the mean-field theory). Combining together with previous works^52,60^ comparing our calculated LDOS and local spectra with the STS measurement, we have obtained a very consistent picture about experimental data for both spectra in real space and in momentum space for the superconducting phase.

Here it is worthwhile to make a special discussion that the nPDW state we chose is among many possible solutions with different periods. Fortunately most of them examined by us have very similar properties except that the periods of modulations could be different. In terms of energy the uniform *d*-wave SC state is the “true” ground state of the *t* − *t*′ − *J* model within our RMFT. However, as we mentioned earlier and before^52^, whenever other weak interactions, such as electron-phonon interaction, nearest neighbor or long range Coulomb force and impurities or defects, are added to the model^53,69^, the nPDW state could be stabilized. Even if we only consider pure *t* − *t*′ − *J* model, these states are in their local minimum. Thus we could study its low energy excitations. At the end of this section, we will discuss the case with different hopping rates *t*_*x*_ and *t*_*y*_ along x and y directions. Then nPDW is stabilized as the ground state if the difference between *t*_*x*_ and *t*_*y*_ is large enough.

Another thing we like to point out is that experiments just like our theory also have found different kinds of CDW states. For the *La*_2−*x*_*Ba*_*x*_*CuO*_4_ family, the period of CDW decreases with doping while it increases for YBCO and BSCCO^11^. These two are called CDW1 and CDW2, respectively, in the review article^37^. There is also CDW3 or the magnetic field induced CDW. In this work we only concentrated on CDW2, which has no magnetic component. We believe CDW1 is probably the stripe state^53^.

When the temperature is raised, the net UPOP in the nPDW state begins to decrease and it becomes negligible at certain temperature *T*_*p*1_. This behavior also supports our assumption that these states are at a local minimum. Then it changes into an IPDW state that still has incommensurate modulations of charge density, bond order and pairing order Δ_*Q*_(**k**) for finite Q and with Δ_0_(**k**) = 0. Magnitude and modulation periods of all these three orders are quite similar to the nPDW state except that the FFT of the charge density does not have a peak at the wave vector of the pairing modulation as seen in nPDW^45^. In IPDW state, the modulation momentum of charge is twice of pairing. These states vary gradually with temperature until it reaches a higher temperature *T*_*p*2_ and there is no longer a solution with modulations of pairing. Quite unexpectedly *T*_*p*2_ actually decreases sharply as doping increases. Figure 5(a) shows that *T*_*p*2_ is proportional to the PG temperature T* with an overestimation of at most a factor two for its values. This is quite satisfactory for a simple mean-field approach like ours. More discussion about this is given below.

Furthermore our analysis shows that the IPDW state near nodal region has a Fermi arc with a fraction of DOS of the full Fermi surface when there is no pairing. There is still a large gap at the antinodal region as shown in Fig. 5(b). The DOS or the length of Fermi arc increases with dopant concentration just as what were seen by ARPES^34^ and NMR^33^.

In our calculation we obtain the uniform *d*-wave SC state at *T* greater than *T*_*p*2_. However as we mentioned earlier, this could be a consequence that we actually are at a local minimum and uniform SC state is the global minimum solution. We believe that if we consider solutions composed of randomly packed x- and y- oriented domains of these IPDW states, its large entropy would have a lower free energy than that of the uniform SC state. Thus the reappearance of the uniform *d*-wave SC state at high *T* is indicating the limit of accuracy of our mean-field theory and it probably has no physical significance. Here we notice that much more accurate numerical work^55^ than our mean-field result for Hubbard model at dopant 0.125 shows that uniform state is not the ground state. The stripes including SDW in addition to PDW and CDW are possible ground states for *U*/*t* = 12 or less. For larger *U* as is for *t* − *J* model, antiferromagnetism is weaker and the nature of ground state is yet to be settled.

It should be emphasized that the IPDW state with finite pairing order Δ_*Q*_(**k**) is also a SC FFLO^39,40^ state with finite momentum pairing if there is a phase coherence. But actually there maybe solutions with disordered fluctuating domains^72^ with different charge density, phases and periods, etc. Variational Monte Carlo calculations have shown^73^ that random stripe domains could be very competitive in energy in comparison with the long-range-ordered state. Furthermore the short-range-ordered domains of these IPDW states will have larger entropy and lower free energy. The PG phase is known to have strong vortex fluctuations^74,75^. The inclusion of phases for these PDW states and their coupling with vortices^45,46,63^ will provide a better and wholistic description of the PG phase. However, the PDW described here, we believe, should still be a basic entity included in these considerations to account for the spectra measured by experiments.

Another important issue we have not addressed is the effect of magnetic field on the PDW. Magnetic field-induced unidirectional CDW states have been reported below and above *T*_*c*_ for YBCO^76–78^. Some are even long-range ordered in 3D^79^. Somewhat different results are found in BSCCO family. Recent experiment on BSCCO has found bidirectional PDW or checkerboard of 8 unit cell period existing inside the vortex halo^80^. For *Bi*_2_*Sr*_2−*x*_*La*_*x*_*CuO*_6_, NMR measurement^81^ shows that an in-plane magnetic field of 10*T* is enough to induce long-range ordered CDW without spin components in the PG phase. Since such a small in-plane field does not have much effect on our nPDW or IPDW states, we believe these states are the ones observed in *Bi*_2_*Sr*_2−*x*_*La*_*x*_*CuO*_6_. This is supported by the good agreement achieved in Fig. 5(c) between the calculated DOS of our IPDW states and the Knight shift measurement in the PG phase by suppressing SC phase with high field^33^.

One of the surprises we found is that without adjustable parameters in our calculations, we are able to get many quantities very close to experimental values and also have very good agreement with very sophisticated numerical works that go much beyond mean-field theory. Considering we are doing a mean-field calculation, this is even more surprising. One main reason could be that the GWA is really effective in catching the strong-correlation physics of the *t* − *J* model. As discussed in our previous paper^52^, the doping dependence of the GW factors are very important in obtaining these solutions. If we set all GW factors to 1, we are not able to find any of these PDW solutions. Based on this premise, we can now provide a very simple picture about the cuprate phase diagrams. Starting at half-filling, the model has the RVB proposed by Anderson^82^ dominate in the Mott insulator. RVB has the *d*-wave pairing and bond order intertwined. But without charge present both of them are actually the variational parameters or hidden orders we defined in Eq. (3). When holes are doped into the lattice, RVB tends to localize the charges to prohibit its fluctuation. Once the localization has failed possibly after the antiferromagnetism is destroyed by the dopant, the system starts to form these unidirectional PDW states which has charge density intertwined with RVB (pairing and bond order). These states have a gap in the antinodal region and in the nodal region a Fermi arc with only a fractional DOS survived. When there is too much doping that these density waves can no longer be viable, then we lose the Mott physics and recovered a FLS^83^. These states then develop an average uniform SC pairing order at lower temperatures although it is relatively small in comparison with large magnitude of pairing modulation. Of course, the phase fluctuation will become more important as temperature rises^37,46^ and mean field results will be revised.

The theory we propose depends on the presence of a PDW state in the PG phase. There is a way to test this hypothesis besides the possibility of using STS^59^, which has to worry about the rapid pairing phase variation in a few lattice spacing and also the measurement being most likely at a higher temperature. For a PDW state in *x*-direction, the magnitude of the gap in the *y*-antinode (0, *π*) is about the same (Gap values near the two antinodes my differ by 10% for different nPDW states. This is probably the accuracy of the mean-filed theory) as the gap in the *x*-antinode (*π*, 0) as shown in Fig. 1. This is contrary to what one would expect if we only have a pure CDW in the *x*-direction. Then the gap opening due to zone folding should be larger in the folding direction. The *x* and *y* asymmetry of the Fermi arc, shown in Fig. 4(f), may be used to distinguish the arc from part of the Fermi pocket^31,32^. We can also examine the particle-hole asymmetry in the PG phase. IPDW will have very similar result as the nPDW state measured by ARPES^35^. Particle-hole asymmetry should be observed away from the Fermi arc. This could be a sign for the presence of finite momentum Cooper pairs^65^.

It has been shown that a magnetic field about 10 *T* is enough to induce a long-range ordered CDW or PDW^79,81^. Since 10 *T* is quite small, it may be possible to generate the long-range order by having a thin tetragonal single layer cuprate deposited on a strained substrate. We have looked at the case with the hopping rate in the *x* direction *t*_*x*_ less than *t*_*y*_ in the *y* direction. The preliminary result shows that the energy of nPDW state for doping concentration *x* = 0.08 is now lower than the uniform *d*-wave SC state if *t*_*x*_ < 0.84 *t*_*y*_. This is consistent with previous work on stripe states at *x* = 0.125^51^. But here we probably overestimate the strength of the uniform state. In real material a small difference between *t*_*x*_ and *t*_*y*_ might be enough to stabilize an IPDW/nPDW. Since the x-directional nPDW has a much lower energy than the y-directional nPDW, the system is likely to be dominated by only x-directional nPDW, and a unidirectional IPDW at *T* > *T*_*c*_. It may also be possible to have a phase coherent IPDW state in a small temperature window that will be a truly new phase. Even without invoking *t*_*x*_ < *t*_*y*_, as shown in Fig. 1, the spectra near (*π*, 0) and (0, *π*) are very different. Now with *t*_*x*_ < *t*_*y*_, the UPOP has the *s*′ + *d* symmetry with pairing in x direction larger than in y. On the other hand, the energy gap near the x-direction antinode (*π*, 0) is getting smaller as strain increases, while the gap near (0, *π*) becomes larger. Thus IPDW in the PG phase could be detected with ARPES in this system.

## Methods

Following the idea of Gutzwiller^58^ and works of Himeda and Ogata^84,85^, we replace the projection operator (*P*_*G*_) in the *t* − *t*′ − *J* Hamiltonian in Eq. (1) with the Gutzwiller renormalization factors. The renormalized Hamiltonian now becomes

2 $$\begin{array}{rcl}H & = & -\,\sum _{i,j,\sigma }\,{g}_{ij\sigma }^{t}{t}_{ij}({c}_{i\sigma }^{\dagger }{c}_{j\sigma }+H.\,C.\,)\\  &  & +\,\,\sum _{\langle i,j\rangle }\,J[{g}_{ij}^{s,z}{S}_{i}^{s,z}{S}_{j}^{s,z}+{g}_{ij}^{s,xy}(\frac{{S}_{i}^{+}{S}_{j}^{-}+{S}_{i}^{-}{S}_{j}^{+}}{2})]\end{array}$$

where ${g}_{ij\sigma }^{t},{g}_{ij}^{s,z}$, and ${g}_{ij}^{s,xy}$ are the Gutzwiller factors, which are dependent on the values of hole density *δ*_*i*_, which is one of our variational parameters along with pair field ${{\rm{\Delta }}}_{ij\sigma }^{v}$ and bond order ${\chi }_{ij\sigma }^{v}$:

3 $$\begin{array}{rcl}{{\rm{\Delta }}}_{ij\sigma }^{v} & = & \sigma \langle {{\rm{\Psi }}}_{0}|{c}_{i\sigma }{c}_{j\bar{\sigma }}|{{\rm{\Psi }}}_{0}\rangle \\ {\chi }_{ij\sigma }^{v} & = & \langle {{\rm{\Psi }}}_{0}|{c}_{i\sigma }^{\dagger }{c}_{j\sigma }|{{\rm{\Psi }}}_{0}\rangle \\ {\delta }_{i} & = & 1-\langle {{\rm{\Psi }}}_{0}|{n}_{i}|{{\rm{\Psi }}}_{0}\rangle \end{array}$$

where |Ψ_0_〉 is the unprojected wavefunction. The superscript *v* is used to denote that these quantities are different from the real physical quantities for comparison with the experiments. The factors are given as

4 $$\begin{array}{rcl}{g}_{ij\sigma }^{t} & = & {g}_{i\sigma }^{t}{g}_{j\sigma }^{t}\\ {g}_{i\sigma }^{t} & = & \sqrt{\frac{2{\delta }_{i}}{1+{\delta }_{i}}}\\ {g}_{ij}^{s,xy(z)} & = & {g}_{i}^{s,xy(z)}{g}_{j}^{s,xy(z)}\\ {g}_{i}^{s,xy(z)} & = & \frac{2}{1+{\delta }_{i}}\end{array}$$

The more general forms of these factors with magnetic moment involved can be found in^51,52,60,86^. In this paper we only consider paramagnetic PDW. We then solve for the BdG equation and perform the iteration until the differences of consecutive variational order parameters are smaller than 10^−3^ and sometime 10^−4^ to verify the *d*-wave order more accurately. For an nPDW state like in Fig. 1, we will have 80 variables to be solved self-consistently.

Because we are going to investigate the features in *k* space, it is necessary to apply the supercell calculation^87^. For each cell we have *N*_*x*_ × *N*_*y*_ sites and the total number of cell is *M*_*c*_ = *M*_*x*_ × *M*_*y*_. Our Hamiltonian is then reduced from 2*M*_*x*_*N*_*x*_ × 2*M*_*y*_*N*_*y*_ to *M*_*x*_ × *M*_*y*_ matrix equation each with lattice size 2*N*_*x*_ × 2*N*_*y*_. The self-consistent solutions now have to be carried out for each cell. The spectral weight can be written with our wave function (*u*, *v*) as:

5 $$\begin{array}{rcl}A(k,\omega ) & = & \frac{1}{N}\,\sum _{ij,n+}\,f(\,-\,{E}_{n})({e}^{i{\bf{k}}\cdot ({{\bf{r}}}_{i}-{{\bf{r}}}_{j})}{g}_{ij\uparrow }^{t}{u}_{i,n}^{{\bf{K}}\ast }{u}_{j,n}^{{\bf{K}}}\delta (\omega -{E}_{n})\\  &  & +\,{e}^{i{\bf{k}}\cdot ({{\bf{r}}}_{j}-{{\bf{r}}}_{i})}{g}_{ij\downarrow }^{t}{v}_{i,n}^{{\bf{K}}}{v}_{j,n}^{{\bf{K}}\ast }\delta (\omega +{E}_{n}))\\  &  & +\,\frac{1}{N}\,\sum _{ij,n-}\,f({E}_{n})({e}^{i{\bf{k}}\cdot ({{\bf{r}}}_{i}-{{\bf{r}}}_{j})}{g}_{ij\uparrow }^{t}{u}_{i,n}^{{\bf{K}}\ast }{u}_{j,n}^{{\bf{K}}}\delta (\omega -{E}_{n})\\  &  & +\,{e}^{i{\bf{k}}\cdot ({{\bf{r}}}_{j}-{{\bf{r}}}_{i})}{g}_{ij\downarrow }^{t}{v}_{i,n}^{{\bf{K}}}{v}_{j,n}^{{\bf{K}}\ast }\delta (\omega +{E}_{n}))\end{array}$$

where **k** = **k**_0_ + **K** while ${k}_{0}=2\pi (\frac{{n}_{x}}{{N}_{x}},\frac{{n}_{y}}{{N}_{y}})$ where ${n}_{x}\in [\,-\,{N}_{x}/2+1,{N}_{x}/2]$, ${n}_{y}\in [\,-\,{N}_{y}/2+1,{N}_{y}/2]$, and $K=2\pi (\frac{{n}_{x}^{c}}{{M}_{x}{N}_{x}},\frac{{n}_{y}^{c}}{{M}_{y}{N}_{y}})$ where ${n}_{x}^{c}\in [0,{M}_{x}-1]$, ${n}_{y}^{c}\in [0,{N}_{y}-1]$. *f*(*E*_*n*_) is the Fermi-Dirac distribution and *n* + (*n*−) means summation over positive (negative) energies. *δ*(*ω* − *E*_*n*_) is the Lorenzian and has the following form:

6 $$\begin{array}{l}\delta (\omega -{E}_{n})=\frac{1}{\pi }\frac{{\rm{\Gamma }}}{{{\rm{\Gamma }}}^{2}+{(\omega -{E}_{n})}^{2}}\end{array}$$

Since a lot of our patterns are unidirectional, we can exploit the translational invariance in y-direction assuming that the modulation is in x-direction to reduce the calculation time. By transforming our original creation/annihilation operators into those with basis of (*i*_*x*_, *k*):

7 $$\begin{array}{l}{c}_{i,\sigma }^{\dagger }=\frac{1}{\sqrt{N}}\sum _{k}{c}_{{i}_{x},\sigma }^{\dagger }(k){e}^{-ik{R}_{{i}_{y}}}\end{array}$$

we could translate our Hamiltonian as in a 1D lattice. With this transformation, we are able to perform the calculation for lattice size two times larger. For the symbols above, *N* represents the lattice size in y-direction, ${R}_{{i}_{y}}$ is the y component of the original lattice vector *i*, and ${c}_{{i}_{x},\sigma }^{\dagger }(k)$ is the creation operator in this quasi-1D system for momentum *k*. Details could be found in ref.^60^.

The UPOP plays an important role in our work. To obtain it, we first calculate $\overline{{{\rm{\Delta }}}_{x}}$ and $\overline{{{\rm{\Delta }}}_{y}}$:

8 $$\begin{array}{l}\overline{{{\rm{\Delta }}}_{x}}=\sum _{{\bf{K}}}\,\sum _{i}^{{N}_{x}}\,{{\rm{\Delta }}}_{ii+\hat{x}}^{{\bf{K}}}/{N}_{x}/{M}_{c}\\ \overline{{{\rm{\Delta }}}_{y}}=\sum _{{\bf{K}}}\,\sum _{i}^{{N}_{x}}\,{{\rm{\Delta }}}_{ii+\hat{y}}^{K}/{N}_{x}/{M}_{c}\end{array}$$

After we obtain the averaged pairing values in x and y direction, we can then calculate UPOP:

9 $$\begin{array}{l}UPOP=\frac{|\overline{{{\rm{\Delta }}}_{x}}|+|\overline{{{\rm{\Delta }}}_{y}}|}{2}\end{array}$$

## Supplementary information

Supplementary materials for: Evolution of Pairing Orders between Pseudogap and Superconducting Phases of Cuprate Superconductors

## Data Availability

The datasets generated during and/or analysed during the current study are available from the corresponding author on reasonable request.

## References

[1] Vojta M (2009). Lattice symmetry breaking in cuprate superconductors: Stripes, nematics, and superconductivity. Adv. Phys..

[2] Keimer B (2015). From quantum matter to superconductivity in copper oxides. Nat..

[3] Shekhter A (2013). Bounding the pseudogap with a line of phase transitions in *YBa*_2_*Cu*_3_*O*_6+*δ*_. Nat..

[4] Zhao L (2017). A global inversion-symmetry-broken phase inside the pseudogap region of *YBa*_2_*Cu*_3_*O*_*y*_. Nat. Phys..

[5] Bourges P, Sidis Y (2011). Novel magnetic order in the pseudogap state of high-T_c_ copper oxides superconductors. Comptes Rendus Physique.

[6] Sato Y (2017). Thermodynamic evidence for a nematic phase transition at the onset of the pseudogap in *YBa*_2_*Cu*_3_*O*_*y*_. Nat. Phys..

[7] Comin R (2015). Broken translational and rotational symmetry via charge stripe order in underdoped *YBa*_2_*Cu*_3_*O*_6+*y*_. Sci..

[8] Wu J, Bollinger AT, He X, Bozovic. (2017). Spontaneous breaking of rotational symmetry in copper oxide superconductors. Nat..

[9] Yamada K (1998). Doping dependence of the spatially modulated dynamical spin correlations and the superconducting-transition temperature in *La*_2−*x*_*Sr*_*x*_*CuO*_4_. Phys. Rev. B.

[10] Abbamonte P (2005). Spatially modulated ‘Mottness’ in *La*_2−*x*_*Ba*_*x*_*CuO*_4_. Nat. Phys..

[11] Comin R, Damascelli A (2016). Resonant x-ray scattering studies of charge order in cuprates. Ann. Rev. Condens. Matter Phys..

[12] Comin R (2015). Symmetry of charge order in cuprates. Nat. Mater..

[13] Yazdani A, Neto EHDS, Aynajian P (2016). Spectroscopic imaging of strongly correlated electronic states. Ann. Rev. Condens. Matter Phys..

[14] Comin R (2014). Charge order driven by fermi-arc instability in *Bi*_2_*Sr*_2−*x*_*La*_*x*_*CuO*_6+*δ*_. Sci..

[15] Torchinsky DH (2013). Fluctuating charge-density waves in a cuprate superconductor. Nat. Mater..

[16] Ghiringhelli G (2012). Long-range incommensurate charge fluctuations in (*Y*, *Nd*)*Ba*_2_*Cu*_3_*O*_6+*x*_. Sci..

[17] Neto EHDS (2014). Ubiquitous interplay between charge ordering and high-temperature superconductivity in cuprates. Sci..

[18] Hashimoto M (2014). Direct observation of bulk charge modulations in optimally doped *Bi*_15_*Pba*_6_*Sr*_154_*CaCu*_2_*O*_8+*δ*_. Phys. Rev. B.

[19] Blanco-Canosa S (2014). Resonant x-ray scattering study of charge-density wave correlations in *YBa*_2_*Cu*_3_*O*_6+*x*_. Phys. Rev. B.

[20] Neto EHDS (2015). Charge ordering in the electron-doped superconductor *Nd*_2−*x*_*Ce*_*x*_*CuO*_4_. Sci..

[21] Kohsaka Y (2007). An intrinsic bond-centered electronic glass with unidirectional domains in underdoped cuprates. Sci..

[22] Kirtley JR (2006). Angle-resolved phase-sensitive determination of the in-plane gap symmetry in *YBa*_2_*Cu*_3_*O*_7−*δ*_. Nat. Phys..

[23] Tsuei CC (2004). Robust *d*_*x*2–*y*2_ pairing symmetry in hole-doped cuprate superconductors. Phys. Rev. Lett..

[24] Warren WW (1989). Cu spin dynamics and superconducting precursor effects in planes above *T*_*c*_ in *YBa*_2_*Cu*_3_*O*_67_. Phys. Rev. Lett..

[25] Timusk T, Statt B (1999). The pseudogap in high-temperature superconductors: An experimental survey. Rep. Prog. Phys..

[26] Marshall DS (1996). Unconventional electronic structure evolution with hole doping in *Bi*_2_*Sr*_2_*CaCu*_2_*O*_8+*δ*_: Angle-resolved photoemmision results. Phys. Rev. Lett..

[27] Loeser AG (1996). Excitation gap in the normal state of underdoped *Bi*_2_*Sr*_2_*CaCu*_2_*O*_8+*δ*_. Sci.

[28] Ding H (1996). Spectroscopic evidence for a pseudogap in the normal state of underdoped high-*T*_*c*_ superconductors. Nat..

[29] Kanigel A (2006). Evolution of the pseudogap from fermi arcs to the nodal liquid. Nat. Phys..

[30] Nakayama K (2009). Evolution of a pairing-induced pseudogap from the superconducting gap of (*Bi*, *Pb*)_2_*Sr*_2_*CuO*_6_. Phys. Rev. Lett..

[31] Doiron-Leyraud N (2007). Quantum oscillations and the fermi surface in an underdoped high-tc superconductor. Nat..

[32] Bangura AF (2008). Small fermi surface pockets in underdoped high temperature superconductors: Observation of shubnikov-de haas oscillations in *YBa*_2_*Cu*_4_*O*_8_. Phys. Rev. Lett..

[33] Kawasaki S (2010). Carrier-concentration dependence of the pseudogap ground state of superconducting *Bi*_2_*Sr*_2−*x*_*La*_*x*_*CuO*_6+*δ*_ revealed by ^63,65^*Cu*-nuclear magnetic resonance in very high magnetic fields. Phys. Rev. Lett..

[34] Hashimoto M (2014). Energy gaps in high-transition-temperature cuprate superconductors. Nat. Phys..

[35] He RH (2011). From a single-band metal to a high-temperature superconductor via two thermal phase transitions. Sci..

[36] Huefner S (2008). Two gaps make a high-temperature superconductor?. Rep. Prog. Phys..

[37] Fradkin E (2015). Colloquium: Theory of intertwined orders in high temperature superconductors. Rev. Mod. Phys..

[38] Lee PA, Nagaosa N, Wen XG (2006). Doping a mott insulator: Physics of high-temperature superconductivity. Rev. Mod. Phys..

[39] Larkin AI, Ovchinnikov YN (1965). Nonuniform state of superconductors. Sov. Phys.-JETP.

[40] Fulde P, Ferrell RA (1964). Superconductivity in a strong spin-exchange field. Phys. Rev..

[41] Podolsky D (2003). A phenomenological theory of the anomalous pseudogap phase in underdoped cuprates. Phys. Rev. B.

[42] Chen HD (2004). Pair density wave in the pseudogap state of high temperature superconductors. Phys. Rev. Lett..

[43] Himeda A, Kato T, Ogata M (2002). Stripe states with spatially oscillating d-wave superconductivity in the two-dimensional *t* − *t*′ − *J* model. Phys. Rev. Lett..

[44] Berg E, Fradkin E, Kivelson S (2009). Theory of the striped superconductor. Phys. Rev. B.

[45] Berg E, Fradkin E, Kivelson S, Tranquada J (2009). Striped superconductors: how spin, charge and superconducting orders intertwine in the cuprates. New J. Phys..

[46] Berg E, Fradkin E, Kivelson S (2009). Charge-4e superconductivity from pair-density-wave order in certain high-temperature superconductors. Nat. Phys..

[47] Sachdev S, Placa RL (2013). Bond order in two-dimensional metals with antiferromagnetic exchange interactions. Phys. Rev. Lett..

[48] Efetov KB, Pepin C (2013). Pseudogap state near a quantum critical point. Nat. Phys..

[49] Wang Y, Chubukov A (2014). Charge-density-wave order with momentum (2*Q*, 0) and (0, 2*Q*) within the spin-fermion model: Continuous and discrete symmetry breaking, preemptive composite order, and relation to pseudogap in hole-doped cuprates. Phys. Rev. B.

[50] Wang Y, Agterberg DF, Chubukov A (2015). Coexistence of charge-density-wave and pair-density-wave orders in underdoped cuprates. Phys. Rev. Lett..

[51] Yang KY (2009). Nature of stripes in the generalized *t* − *j* model applied to the cuprate superconductors. New J. Phys..

[52] Tu WL, Lee TK (2016). Genesis of charge orders in high temperature superconductors. Sci. Rep..

[53] Chou CP, Lee TK (2010). Mechanism of formation of half-doped stripes in underdoped cuprates. Phys. Rev. B.

[54] Corboz P, Rice TM, Troyer M (2014). Competing states in the *t* − *j* model: uniform d-wave state versus stripe state. Phys. Rev. Lett..

[55] Zheng BX (2017). Stripe order in the underdoped region of the two-dimensional hubbard model. Sci..

[56] Capello M, Raczkowski M, Poilblanc D (2008). Stability of rvb hole stripes in high-temperature superconductors. Phys. Rev. B.

[57] Raczkowski, M. *et al*. Unidirectional d-wave superconducting domains in the two-dimensional t−J model. *Phys. Rev. B* **76**, 140505(R) (2007).

[58] Gutzwiller M (1963). Effect of correlation on the ferromagnetism of transition metals. Phys. Rev. Lett..

[59] Hamidian MH (2016). Detection of a cooper-pair density wave in *Ba*_2_*Sr*_2_*CaCu*_2_*O*_8+*x*_. Nat.

[60] Choubey P, Tu WL, Lee TK, Hirschfeld PJ (2017). Incommensurate charge ordered states in the *t* − *t*′ − *J* model. New J. Phys..

[61] Fujita K (2014). Direct phase-sensitive identification of a d-form factor density wave in underdoped cuprates. Proc. Nat. Acad. Sci..

[62] Berg E (2007). Dynamical layer decoupling in a stripe-ordered high-*T*_*c*_ superconductor. Phys. Rev. Lett..

[63] Agterberg D, Tsunetsugu H (2008). Dislocations and vortices in pair-density-wave superconductors. Nat. Phys..

[64] Baruch S, Orgad D (2008). Spectral signatures of modulated *d*-wave superconducting phases. Phys. Rev. B.

[65] Lee PA (2014). Amperean pairing and the pseudogap phase of cuprate superconductors. Phys. Rev. X.

[66] Vishik IM (2012). Phase competition in trisected superconducting dome. Proc. Nat. Acad. Sci..

[67] Mesaros A (2016). Commensurate 4a0-period charge density modulations throughout the *Bi*_2_*Sr*_2_*CaCu*_2_*O*_8+*x*_ pseudogap regime. Proc. Nat. Acad. Sci..

[68] McMillan WL (1976). Theory of discommensurations and the commensurate-incommensurate charge-density-wave phase transition. Phys. Rev. B.

[69] Chou CP, Lee TK (2012). Inhomogeneous state of the extended *t* − *j* model on a square lattice: A variational monte carlo and gutzwiller approximation study. Phys. Rev. B.

[70] Yang KY (2006). Low-energy physical properties of high-tc superconducting cu oxides: A comparison between the resonating valence bond and experiments. Phys. Rev. B.

[71] Alldredge JW (2008). Evolution of the electronic excitation spectrum with strongly diminishing hole density in superconducting *Bi*_2_*Sr*_2_*CaCu*_2_*O*_8+*δ*_. Nat. Phys..

[72] Huang EW (2017). Numerical evidence of fluctuating stripes in the normal state of high-*t*_*c*_ cuprate superconductors. Sci..

[73] Chou CP, Fukushima N, Lee TK (2008). Cluster-glass wave function in the two-dimensional extended *t* − *j* model. Phys. Rev. B.

[74] Xu ZA, Ong NP, Wang Y, Kakeshita T, Uchida S (2000). Vortex-like excitations and the onset of superconducting phase fluctuation in underdoped *La*_2−*x*_*Sr*_*x*_*CuO*_4_. Nat..

[75] Anderson, P. W. Last words on the cuprates. *arXiv*: 1612.03919 (2016).

[76] Chang J (2012). Direct observation of competition between superconductivity and charge density wave order in *YBa*_2_*Cu*_3_*O*_6.67_. Nat. Phys..

[77] Wu T (2013). Emergence of charge order from the vortex state of a high-temperature superconductor. Nat. Comm..

[78] Blanco-Canosa S (2013). Momentum-dependent charge correlations in *YBa*_2_*Cu*_3_*O*_6+*δ*_ superconductors probed by resonant x-ray scattering: Evidence for three competing phases. Phys. Rev. Lett..

[79] Gerber S (2015). Three-dimensional charge density wave order in *YBa*_2_*Cu*_3_*O*_6.67_ at high magnetic fields. Sci..

[80] Edkins, S. D. *et al*. Magnetic-field induced pair density wave state in the cuprate vortex halo. *arXiv*: 1802.04673 (2018). 10.1126/science.aat1773 31171694

[81] Kawasaki S (2017). Charge-density-wave order takes over antiferromagnetism in *Bi*_2_*Sr*_2−*x*_*La*_*x*_*CuO*_6_ superconductors. Nat. Comm..

[82] Anderson PW (1987). The resonating valence bond state in *La*_2_*CuO*_4_ and superconductivity. Sci..

[83] Badoux S (2016). Change of carrier density at the pseudogap critical point of a cuprate superconductor. Nat..

[84] Himeda A, Ogata M (1999). Coexistence of *d*_*x*2–*y*2_ superconductivity and antiferromagnetism in the two-dimensional *t* − *j* model and numerical estimation of gutzwiller factors. Phys. Rev. B.

[85] Ogata M, Himeda A (2003). Superconductivity and antiferromagnetism in an extended gutzwiller approximation for *t* − *j* model: effect of double-occupancy exclusion. J. Phys. Soc. Jpn..

[86] Christensen RB, Hirschfeld PJ, Anderson BM (2011). Two routes to magnetic order by disorder in underdoped cuprates. Phys. Rev. B.

[87] Schmid M (2010). d-wave superconductor as a catalyst for antiferromagnetism in underdoped cuprates. New J. Phys..

