# Supplementary material for: Evolution of Pairing Orders between Pseudogap and Superconducting Phases of Cuprate Superconductors
Source: Sci Rep. 2019 Feb 8;9:1719. doi: 10.1038/s41598-018-38288-7 (PMC6368576; doi:10.1038/s41598-018-38288-7)
Supplement: Supplementary file 1 — Supplementary materials for: Evolution of Pairing Orders between Pseudogap and Superconducting Phases of Cuprate Superconductors [file 41598_2018_38288_MOESM1_ESM.pdf]

Supplementary materials for: Evolution of Pairing Orders between Pseudogap and Superconducting Phases of Cuprate Superconductors

Wei-Lin Tu[1,2,3] and Ting-Kuo Lee[3,*]

[1]Department of Physics, National Taiwan University, Daan Taipei 10617, Taiwan
[2]Laboratoire de Physique Théorique, IRSAMC, Université de Toulouse, CNRS, UPS, France
[3]Institute of Physics, Academia Sinica, Nankang Taipei 11529, Taiwan
[*]tklee@phys.sinica.edu.tw

ABSTRACT

In this supplementary material we will discuss some details left out from the main article due to the limit of length. The structure is as the following: In section **1**, we will revisit the nPDW and show more results of this exotic state. We will also discuss the discommensurate(DC) patterns first proposed by Mesaros *et al.* [3]. Then, we head to answer some details only briefly mentioned in the main text in section **2**. Section **2.1** will explain how we determined k_G by the usage of EDCs. Section **2.2** will discuss again the two-gap plots shown in Fig. 2 and 5b in the main text, aiming to show readers that these plots are free from parameter(Γ) chosen. Section **2.3** will analyze the effect of Γ in our results. At last, section **2.4** will revisit the Fermi arc and LDOS for our patterns to help distinguish between nPDW and lPDW.

1 Various PDW states including discommensurate PDW

The nodal pair density wave(nPDW) state first proposed by Tu and Lee [1] comes from the anti-phase charge density wave(AP-CDW) state but with a non-zero uniform pairing order parameter(UPOP), which is generated from its (quasi-)incommesurate nature. This accords with previous experimental data [4] that within the superconducting dome, the modulations of Cu-O surface observed are incommensurate. Fig. S1 shows some basic characteristics of nPDW in a 32×32 lattice size. Fig. S1(a) shows that the hole density is maximum at the domain walls near sites 2,7,10 and 15. For the Fourier transform in Fig. S1(b) and S1(c), it is clear that although there are several peaks, the leading one is the one at $\pi/2(\pi/4)$ for hole density(pairing order), which corresponds to the modulation of $4a_0(8a_0)$. Fig. S1(d) demonstrates the local density of state(LDOS) of several chosen sites and the v-shape near zero energy indicates a d-wave pairing gap with a node is opened. Finally, in Fig. S1(e) the comparison of different form factors confirms the dominance of d form factor [2].

McMillan [5] was the first to define a “discommensuration”(DC) as a defect in a commensurate CDW state. In such state, the phase of the CDW jumps between discrete lattice-locked values. Mesaros *et al.* [3] showed that this kind of CDW could be what was observed by experiments. Hence, let us consider a sinusoidal modulation in one spatial dimension with $4a_0$ modulation but a phase jump between each domain, it can be written as:

$$\psi(x) = Aexp[i(Q_0 x+\phi)] \tag{S8}$$

where A is the amplitude and $Q_0 = 4a_0$. The additional phase ϕ defines the phase shift for each domain. For example, Fig. S2(a) shows the modulation in x-direction for one of the DC patterns we have obtained. We will name it after the discommensurate nPDW state(DCnPDW). It is clear that there are two separate domains, one with pink color(sites 0-3, 12-15, and 24-27) with $\phi = 0$ and the other with Green color(sites 6-9, 18-21, and 30-33) with $\phi = \pi$. Moreover, in Fig. S2(b)-(d) we can see its FT shows that the averaged modulation is no longer $4a_0$. This might explain for the reason why there are some experiments which ended up observing the modulation period of $4a_0$ but the others with incommensurability. They can originate from the same phase with local $4a_0$ feature but a global incommensurability. Even with the discommensurability, however, they still possess the same dominant symmetry. As shown in Fig. S2(b), the leading form factor is still d form.

One of the most important points we need to clarify is that despite the nPDW and DCnPDW already mentioned, we can easily obtain a number of different states by changing the initial inputs or lattice size. Each of them has slightly different values of pairing, charge density and bond orders. Fig. S3(a) lists some of the examples and demonstrates their energies. One can see that in fact their energies are nearly degenerate, as claimed previously [1], although the lattice size is different. Even with the same lattice size, it is also possible to have two distinct patterns, as shown in Fig. S3(a). Within the states shown, there are two

of them labeled with QIAPCDW, which is the abbreviation of quasi-incommensurate anti-phase CDW. Different from nPDW, this pattern has zero UPOP, just like AP-CDW in ref. [1]. Amazingly, even though QIAPCDW and nPDW seem very different because of the existence of UPOP, these two still share nearly degenerate energies. This suggests what we have claimed in ref. [1] that in fact all the orders(Δ, χ, etc.) are, instead of competing, intertwined and influencing each other. That is why such different states can possess nearly the same energy. We have to make it clear that the patterns listed here are only some of the possibilities and in fact there can be many more different states. Moreover, we like to point out that these QIAPCDW states are quite similar with the IPDW states, except the latter is generated by raising the temperature of the nPDW states. Except for their energies, there are also some characteristics which these states all share with. One of them is the d form factor symmetry. Among all these states, surprisingly, they all have leading d form factor over s and s', which is one of the important feature of nPDW. In Fig. S3(b), we have collected the values of magnitude of d form factor for each states. We can see although for different states their magnitudes of d form factor vary from each other, most values are within the range from 0.15 to 0.2. Moreover, the ending points are all within the range of 0.18∼0.22, which is very near the quantum critical point observed by experiment around 0.19.

Except for the usual FFT, one of the central goals of this work is to investigate the properties of patterns found in k space. Among all, the most important one is the quasiparticle spectra. In Fig. S4, we demonstrate the spectra at $(\pi,0)$ and $(0,\pi)$ for three different patterns chosen: nPDW at 30 and 32 lattice size, and DCnPDW at 36 lattice size. It is clear that, as mentioned in the main text, the spectra at antinodes $(\pi,0)$ and $(0,\pi)$ are quite different but the gap values are very similar [6]. More interestingly, although there are three different states, their quasi-particle spectra are also very close to each other. Even we increase the temperature, there is still not qualitative difference for the spectra, but only that the gap values decrease a bit as temperature rises. One need to note that at $T = 0.035t$ and $T = 0.05t$ the states have already evolved to IPDW states. Once again, this result suggests that although there are numerous possibilities of having a commensurate, (quasi-)incommensurate, or discommensurate state w/wo UPOP, the deeper cause is alway the same: strong correlated Mott physics with the Gutzwiller factors.

2 Other discussions

In this section, we shall discuss several details left out in the main text, such as the method we used to determined k_G, the two gaps and Fermi arcs. We will also discuss the effect of using different Γ in calculating the spectra density.

2.1 Method to determine k_G

We have mentioned in the main text that as the cut of spectra goes away from the node toward near the antinode, the momentum of gap(k_G) will also deviate from k_F. The way of determining k_G will require the usage of energy distribution curves(EDCs). In Fig. S5(b), we show the spectra at $(\pi,0)$ for nPDW in the doping level of 0.15(30×30 lattice size). Next to the spectra, we also put in a series of EDC cuts starting from the point $(\pi,0)$ toward (π,π). Just like the ARPES experiment, we can easily determine k_G when the minimum gap is reached by looking at the EDC cuts. Note that here we use $\Gamma = 0.25|E|$. The difference between k_G and k_F as a function of doping is shown in Fig. S5(a). The difference becomes smaller as doping increases. This is expected since the gap approaches a pure d-wave gap as doping increases and particle-hole symmetry is recovered for the usual BCS superconductors.

2.2 Two-gap plots

Here we discuss the method used to determine gap values in Fig. 2 and 5(b) in the main text, as well as their error bars. First we will explain that in fact there is only small difference if we utilize different ways of determining gap. In the main text, all the values are determined by using EDCs and the horizontal error bars come from the finite size effect, which could be reduced if we further apply supercells with larger size, while the vertical error bars come from either the width of peaks(due to the choice of Γ), or the fact that there are actually several peaks coexisting. But in fact there are different ways of determining gap values and they will provide the same outcomes. For example, the quasiparticle spectra can be also used to determine the gap as explained in the main text. The result are all the same no matter which way we decided to exploit. Fig. S6(a) put together two curves of gap values determined by EDC and quasiparticle spectra. One can see that these two lines are very close and even if there are small differences, they are within the error bars.

2.3 Choices of Γ

In the main text, we mentioned that the width Γ is chosen as different values for better demonstration in different plots. But in fact we have done a series of analysis showing that there is no qualitative difference in choosing Γ to be a constant as $0.01t$ or as $0.25\sqrt{E^2+T^2}$. In Fig. S6(b) we plotted the same figure as Fig. 5(c) in the main text. But here we include also the curve using $\Gamma = 0.01t$. One can see clearly that there is only small quantitative differences between two blue curves. Our second proof is to investigate the two-gap plots as Fig. 2 and 5(b) in the main text, with different choice of Γ. In Fig. S6(c) we show

the curves of gap values for nPDW at $\delta = 0.15$, but under different choices of Γ. We can see that those three curves are nearly the same within error bars.

Last but not least, we also need to check the consistency of quasi-particle spectra. In Fig. S6(d) and (e), we plotted the same spectra but with different Γ, one with $\Gamma = 0.01t$(d) and another with $\Gamma = 0.25|E|$(e). If we discount the broadening of Fig.S6(e), 6(d) and 6(e) have the same k_G.

2.4 Fermi arcs and LDOS

We have shown in the main text that the UPOP of nPDW is decreasing when temperature rises. The resulting pattern is called IPDW by us, which is also a PDW phase but UPOP is close to zero. Fig. 4(f) in the main text plots the zero energy quasiparticle spectra weight in momentum space and it reveals the feature of the so-called Fermi arc. However, in experiments arcs usually have x and y rotational symmetry. That is because the experimental detection scans over a region of materials that contains domains with modulations in both x and y direction. Therefore the resulting arcs would have the rotational symmetry. In order to compare with their results, we took average of x and y axis of our arcs and replotted it. The resulting figure is as Fig. S7(b), which looks more like the experimental data.

One of the main differences upon having UPOP or not is to look at the LDOS. Since our nPDW possesses d-wave UPOP, its LDOS will have a v-shape feature near the Fermi energy. However, for IPDW there is no UPOP and therefore the DOS at Fermi energy should be non-zero. Consequently, to further confirm the vanishing UPOP, we compare the LDOS of sites near and away from domain walls in Fig. S7(c) and S7(d), respectively. LDOS for five different temperatures are shown and the state remains nPDW for $T = 0$ and $47K$ but becomes IPDW at $T = 94K$, $163K$, and $232K(0.1t \sim 464K)$ because of the disappearance of UPOP. According to the LDOS plots, it is also clear that the v-shape feature disappears gradually as temperature rises, confirming that the node has changed into an arc in IPDW state.

Reference

1. Tu, W. L. and Lee, T. K.. Genesis of charge orders in high temperature superconductors. *Sci. Rep.* **6**, 18675 (2016).
2. Choubey, P., Tu, W. L., Lee, T. K., & Hirschfeld, P. J.. Incommensurate charge ordered states in the $t - t' - J$ model. *New J. Phys.* **19**, 013028 (2017).
3. Mesaros, A. *et al.*. Commensurate $4a_0$-period charge density modulations throughout the $Bi_2Sr_2CaCu_2O_{8+x}$ pseudogap regime. *Proc. Nat. Acad. Sci.* **113**, 12661 (2016).
4. Hamidian, M. H. *et al.*. Atomic-scale electronic structure of the cuprate d-symmetry form factor density wave state. *Nature Phys.* **12**, 150 (2015).
5. McMillan, W. L.. Theory of discommensurations and the commensurate-incommensurate Charge-density-wave phase transition. *Phys. Rev. B* **14**, 1469-1502(1976).
6. Gap values near the two antinodes my differ by 10% for different nPDW states. This is probably the accuracy of the mean-field theory.

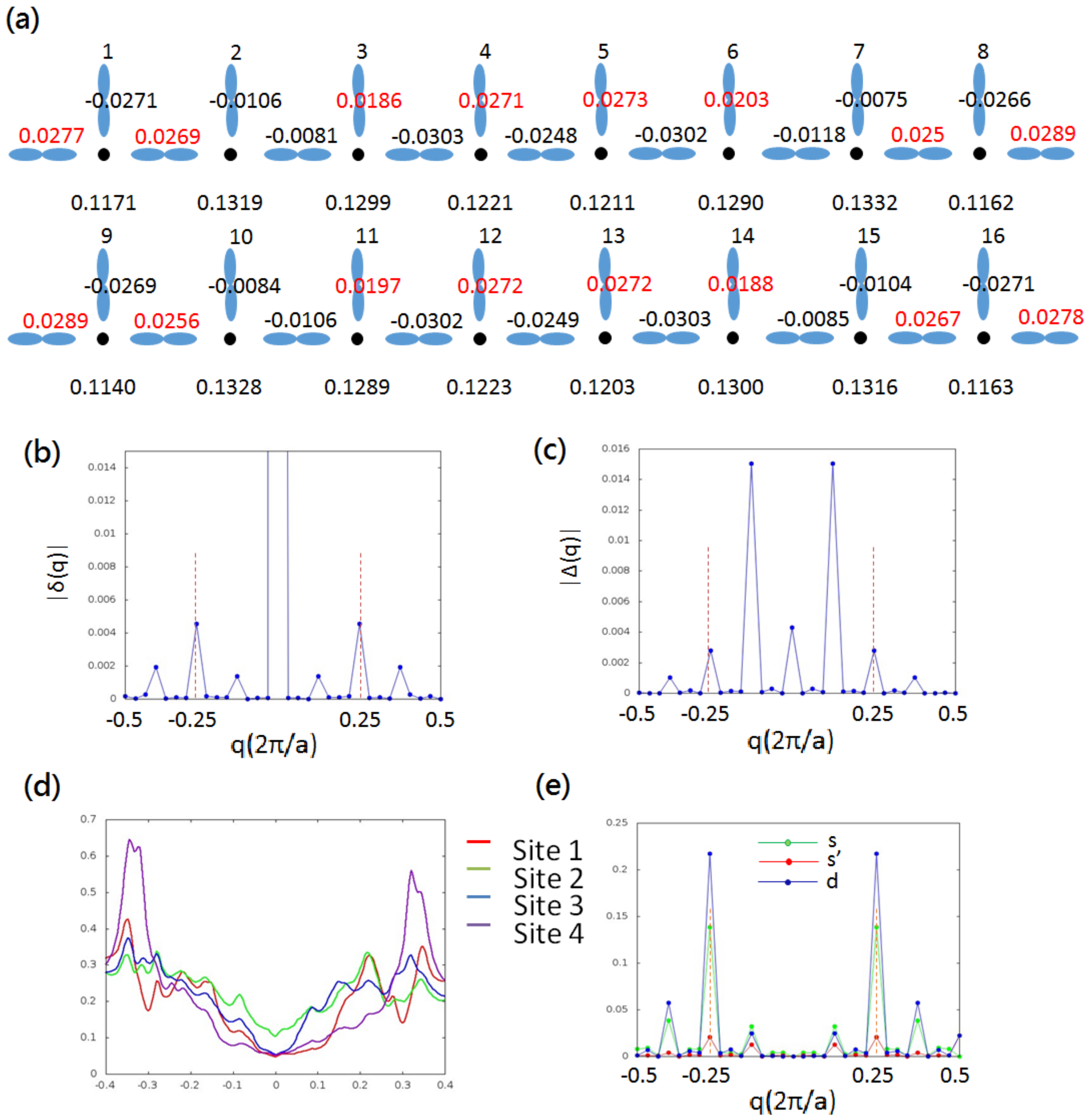

Fig. S 1. Properties of nPDW. (a) The real space modulation of nPDW in 32×32 lattice sites with $\delta = 0.125$. Since the pattern repeats itself with an inversion symmetry in the middle bond, here we only show the first 16 sites. The red and black numbers on each bond denote the values of pairing order and the number at each site (black dots) is the hole density. (b)(c) The Fourier transform of the value of hole density(b) and pairing order(c). (d) LDOS of the first 4 sites of this 32×32 nPDW. (e) Different form factors.

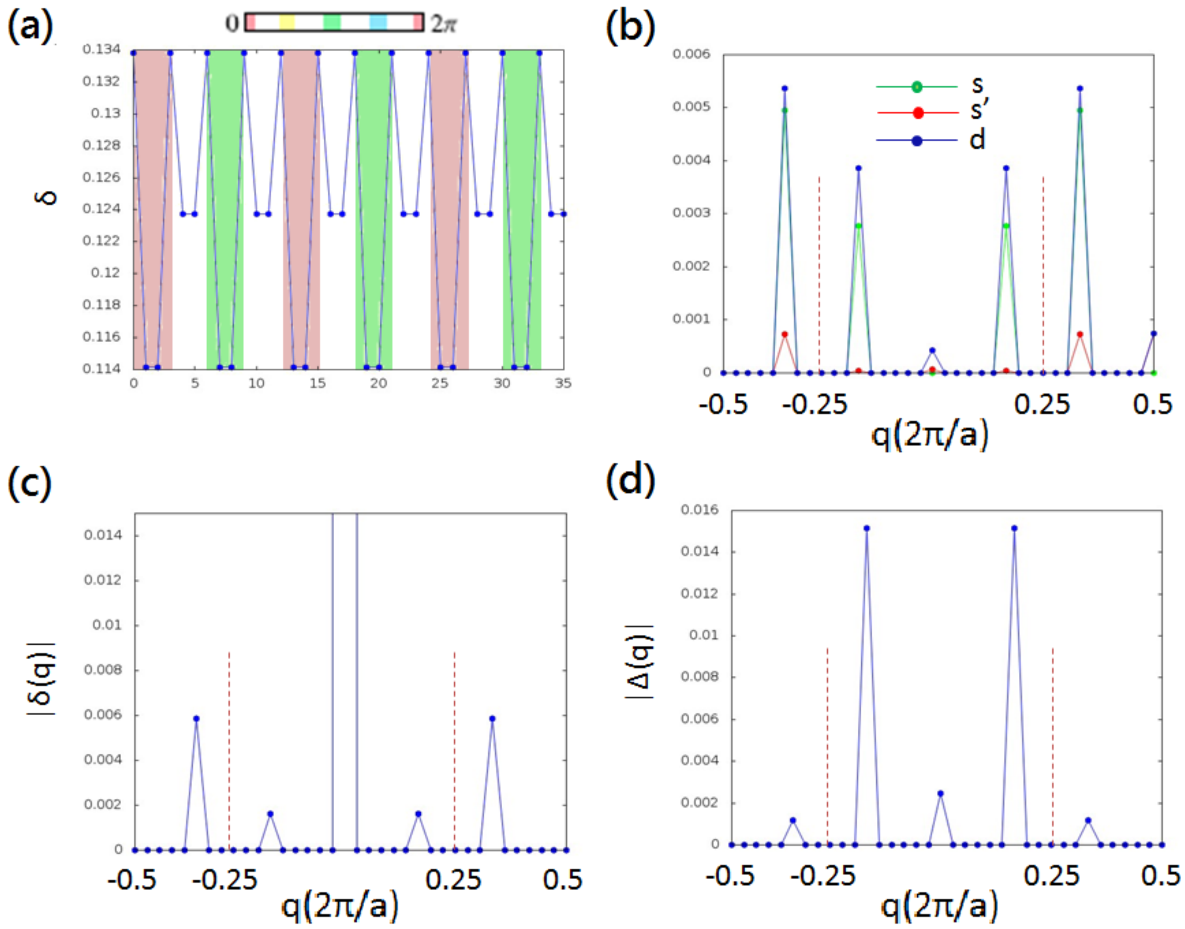

Fig. S 2. Figures showing the properties of discommensurate nPDW. (a) The phase variation of this pattern. Site 0-3, 12-15, and 24-27 are of phase equal to $0(2\pi)$ while sites 6-9, 18-21, and 30-33 are of phase π. (b) Form factors for discommensurate nPDW. We also include the Fourier transform of hole density(c) and pairing order(d).

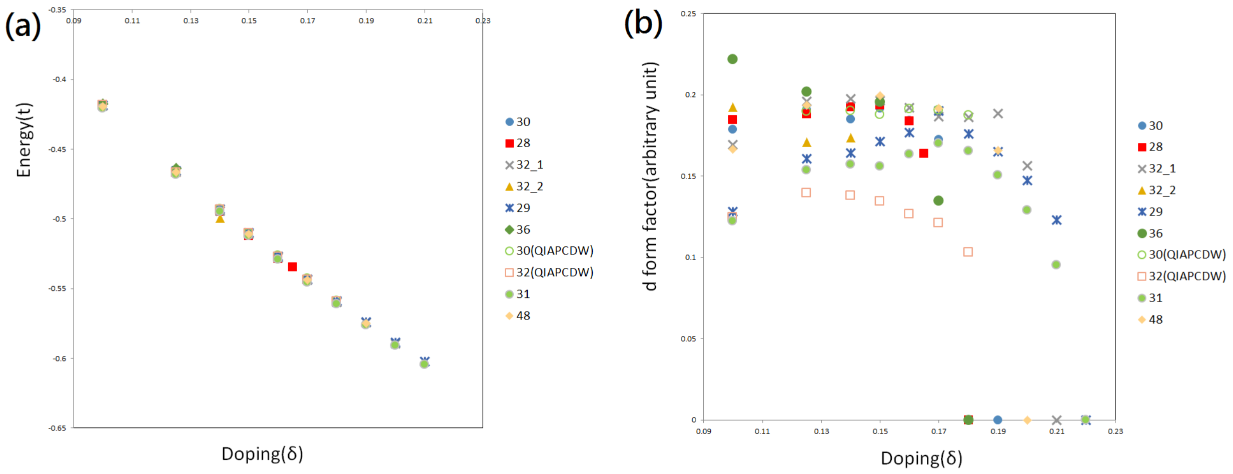

Fig. S 3. (a) Energies of several states chosen by us. Although we have listed ten different states here, their energies seem to be nearly degenerate and follow the same trend line. (b) Magnitude of d form factor of patterns. Given different states we expect their magnitude to change but still all of them seem to have the same trend: the magnitude maintains the same until doping level exceeds 0.18, where it starts to drop drastically and becomes zero in the range of 0.18∼0.22.

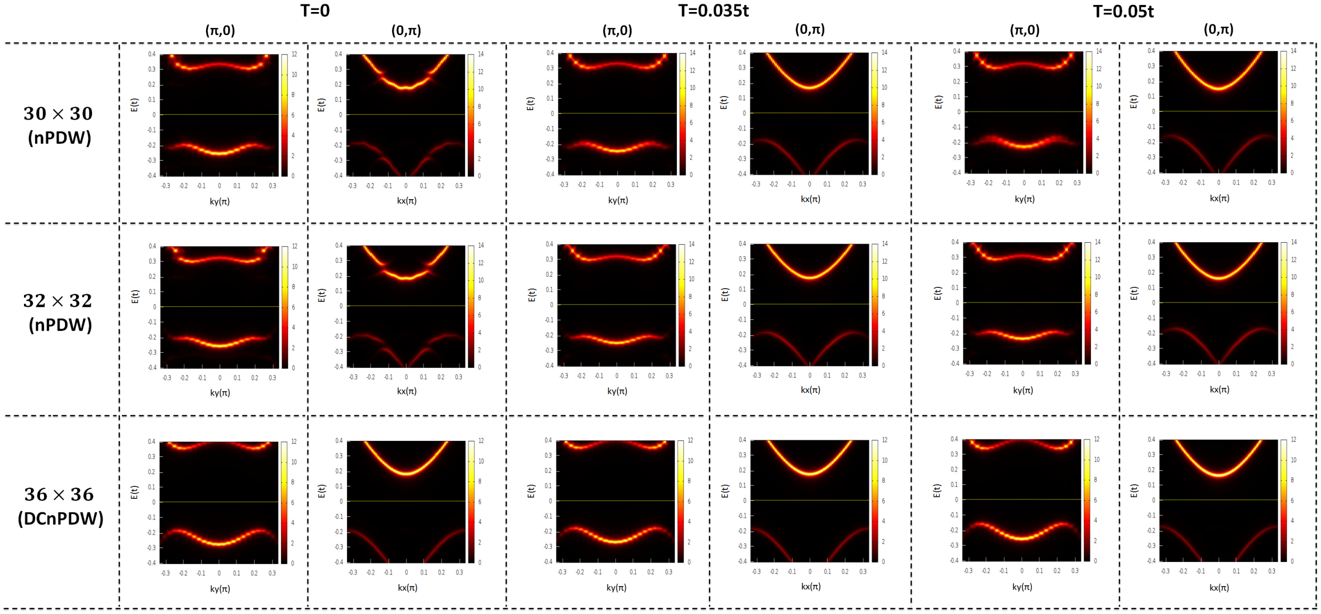

Fig. S 4. We list several quasi-particle spectra at antinodes($(\pi,0)/(0,\pi)$) for three different patterns at different temperatures. Although marked as nPDW in the first column, the patterns become IPDW at $T = 0.035t$ and $T = 0.05t$. However their spectra do not change much and the differences of gap values at $(\pi,0)$ and $(0,\pi)$ are within 10% [6].

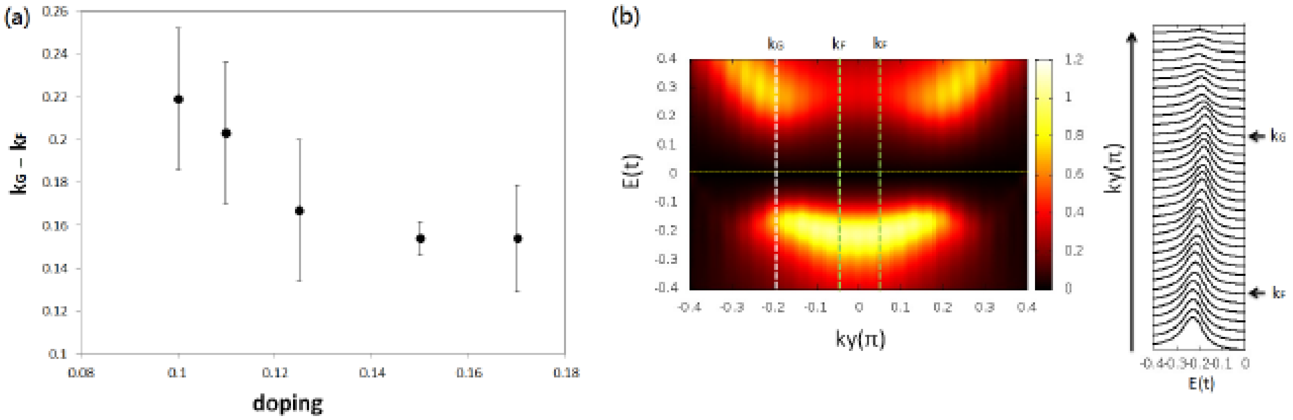

Fig. S 5. (a) A collection of several data points of $k_G - k_F$ vs doping at $k_x = \pi$. The way of determining the difference of k_G and k_F is shown in (b): k_G determined by examining EDCs plotted from $k_y = 0$ toward $k_y = pi$, for dopant concentration 0.15. k_F is determined by Fermi liquid surface and marked along with k_G on the EDC plot. The quasiparticle spectra is also shown with Gaussian width $\Gamma = \alpha|E|$ ($\alpha = 0.25$) and marked with positions of k_G and k_F.

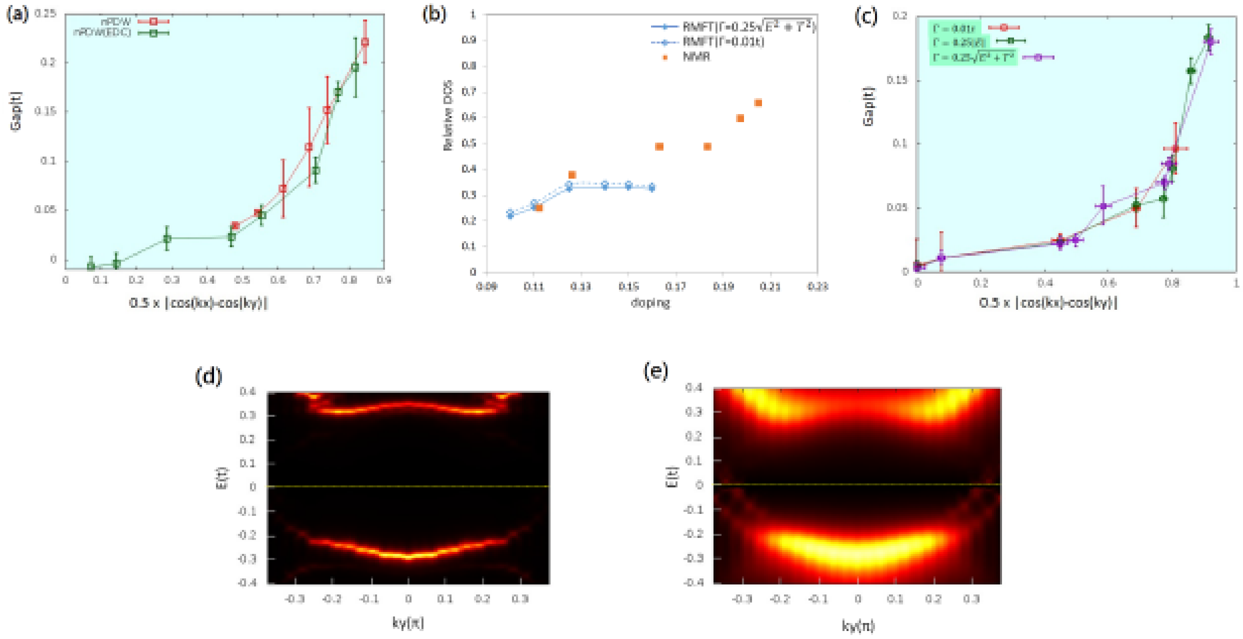

Fig. S 6. (a) Two-gap plot for nPDW at $\delta = 0.125$ as shown in Fig. 3 in the main text but obtained from different approaches: red line is determined by the gap values shown by quasiparticle spectra but green line comes from EDCs, the same way as in main text. (b) Relative DOS as a function of hole concentration as in Fig.7(b) in the main text but put together with two different Γ. The two blue lines are very close to each other. (c) Two gap plots determined by different Γ for nPDW at $\delta = 0.15$. One can see that these lines nearly overlap with each other. Figure (d) and (e) again show the quasi-particle spectra for nPDW at $\delta = 0.125$(for the 32×32 lattice) at $k_x = 0.977\pi$ but with different Γ: (d) $\Gamma = 0.01t$ and (e) $\Gamma = 0.25|E|$. Note that in fact (d) is identical as Fig. 1(f) in the main text. We can find that although these two figures look quite different due to the choices of Γ, important features such as location of k_G are still the same, only that in (e) the spectra bands are broadened due to larger Γ.

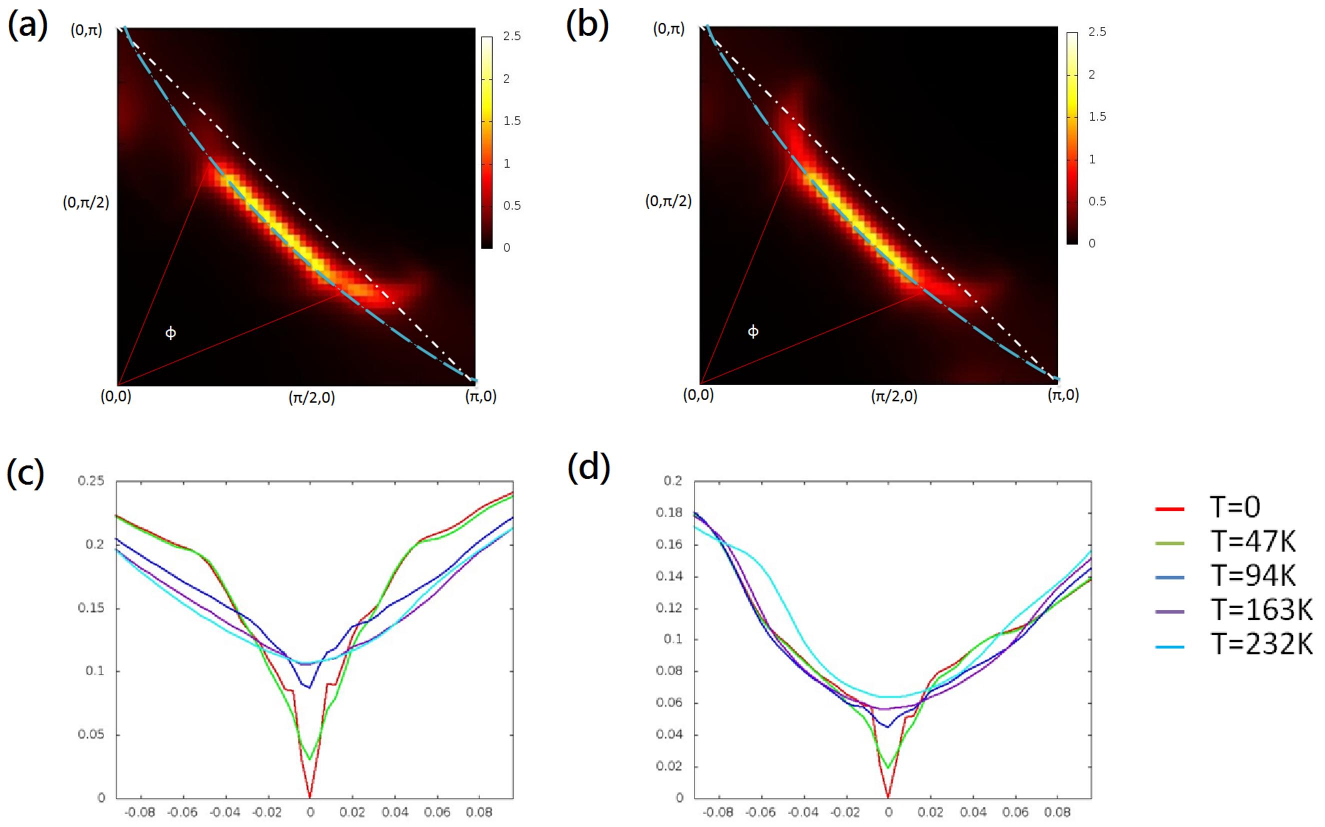

Fig. S 7. (a) and (b) Zero energy quasiparticle spectra in k space before(a) and after(b) taking average of x- and y-directions PDW. (a) is the same as Fig. 4f in the main text and we put it here again for the reason of comparison. Clearly, (b) looks more like the observation by experimental groups. (c) and (d) LDOS at sites near(c) and away from(d) domain walls at different temperatures for nPDW(IPDW) at $\delta = 0.15$. Γ used here is equal to $\alpha\sqrt{E^2+T^2}(\alpha = 0.25)$. All figures shown here are of 30×30 lattice size. Its T_{p1} is around $90K$.
